# Butanolides and Butenolides from a Marine-Derived *Streptomyces* sp. Exert Neuroprotective Activity through Activation of the TrkB Neurotrophin Receptor

**DOI:** 10.3390/md21090465

**Published:** 2023-08-25

**Authors:** Paolo Giaccio, Despoina Charou, Dafni-Ioanna Diakaki, Anna Chita, Achille Gravanis, Ioannis Charalampopoulos, Vassilios Roussis, Efstathia Ioannou

**Affiliations:** 1 Section of Pharmacognosy and Chemistry of Natural Products, Department of Pharmacy, National and Kapodistrian University of Athens, Panepistimiopolis Zografou, 15771 Athens, Greece; pgiaccio@pharm.uoa.gr (P.G.); dafnid@pharm.uoa.gr (D.-I.D.); roussis@pharm.uoa.gr (V.R.); 2 Department of Pharmacology, Medical School, University of Crete, 71003 Heraklion, Greece; dcharou@gmail.com (D.C.); anna.chita2@gmail.com (A.C.); gravanis@med.uoc.gr (A.G.); charalampn@uoc.gr (I.C.); 3 Institute of Molecular Biology & Biotechnology, Foundation for Research & Technology-Hellas (IMBB-FORTH), 70013 Heraklion, Greece

**Keywords:** marine actinobacteria, *Streptomyces*, γ-lactones, neurodegenerative diseases, neuroprotection, TrkB receptor, BDNF

## Abstract

Neurodegenerative diseases are incurable and debilitating conditions, characterized by progressive loss and degeneration of vulnerable neuronal populations. Currently, there are no effective therapies available for the treatment of most neurodegenerative disorders. A panel of extracts exhibiting interesting chemical profiles among a high number of bacterial strains isolated from East Mediterranean marine sediments and macroorganisms were evaluated for their activity on TrkB-expressing cells. Among them, the actinobacterial strain *Streptomyces* sp. BI0788, exhibiting neuroprotective activity in vitro, was selected and cultivated in large-scale. The chemical analysis of its organic extract resulted in the isolation of four new butanolides (**1**, **4**–**6**), along with two previously reported butanolides (**2** and **3**) and eight previously reported butenolides (**7**–**14**). Compounds **2**–**4** and **7**–**14** were evaluated for their neuroprotective effects on TrkB-expressing NIH-3T3 cells. Among them, metabolites **3**, **4**, **7**, **10**, **11**, **13** and **14** exhibited significant protective activity on the aforementioned cells through the activation of TrkB, the high-affinity receptor for the Brain-Derived Neurotrophic Factor (BDNF), which is well known to play a crucial role in neuronal cell survival and maintenance.

## 1. Introduction

Currently there is an urgent need to identify and develop new therapeutics for the treatment of neurodegenerative diseases [1]. Neurodegenerative diseases, such as Alzheimer’s disease, Parkinson’s disease, Huntington’s disease, spinal muscular atrophy, multiple sclerosis and motor neuron disease, are neuropathological conditions characterized by progressive structural and, consequently, functional degeneration of the nervous system that is essential for mobility, coordination, strength, sensation and cognition [2,3]. Neurodegenerative disorders are the third most common cause of disability and premature death in the EU, and their prevalence is likely to increase as the European population ages [4]. Even though there are a few drugs prescribed to alleviate certain symptoms, there are limited or no approved therapies for the treatment of these neuropathological conditions. One of the most promising therapeutic approaches against neurodegenerative progression is the application of neurotrophins, endogenous growth factors that effectively protect neural cells against toxic products of degenerating tissues, while promoting neuronal repair and regeneration. Among them, the Brain-Derived Neurotrophic Factor (BDNF) and its high-affinity receptor TrkB are key players for neuronal survival, differentiation and circuit maintenance under physiological and neuropathological conditions [5,6].

Over the last years, the marine environment has been recognized as an ecosystem hosting unique taxa of microorganisms, especially actinobacteria [7], which are underexploited, even though they have shown great potential for drug development [8,9,10]. Lately, interesting compounds of different chemical classes, such as polyketides, peptides, macrolides, indoles, aminoglycosides and terpenes, exhibiting a wide range of therapeutical properties, including antibacterial, antiviral, anticancer, and immunosuppressive activities, have been reported from marine actinobacterial strains [9,11,12,13].

**Figure 1:**
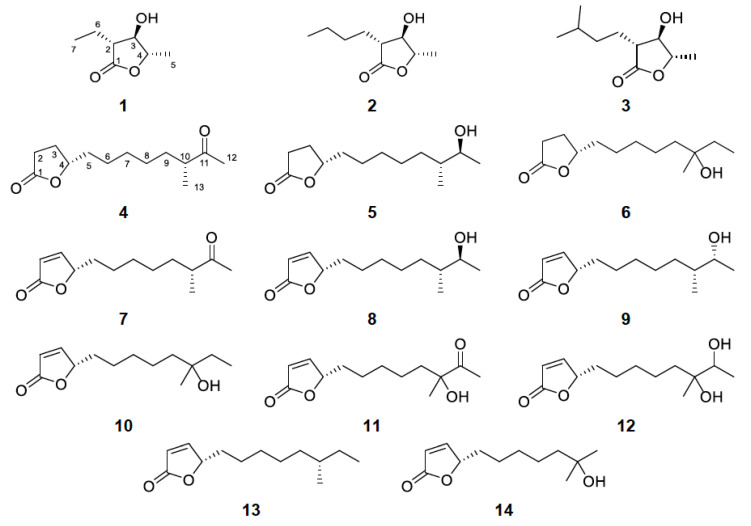
Chemical structures of compounds **1**–**14** isolated from the actinobacterial strain *Streptomyces* sp. BI0788.

In search of new molecules with neuroprotective activity, several extracts exhibiting interesting chemical profiles among a high number of bacterial strains isolated from East Mediterranean marine sediments and macroorganisms were evaluated for their activity on the TrkB-stably expressing cell line NIH-3T3 (NIH-3T3-TrkB). Among them, the actinobacterial strain BI0788, exhibiting protective activity in vitro, was selected for further chemical investigation. Extraction of a large-scale culture of the bacterial strain BI0788 and fractionation of the resulting organic residue allowed for the isolation of fourteen γ-lactone-containing metabolites (**1**–**14**, Figure 1), including four new natural products (**1** and **4**–**6**).

Herein, we report the isolation and structure elucidation of the new butanolides **1** and **4**–**6**, as well as the evaluation of the protective activity of compounds **2**–**4** and **7**–**14** on NIH-3T3-TrkB cells through selective activation of the TrkB receptor.

## 2. Results

**Figure 2:**
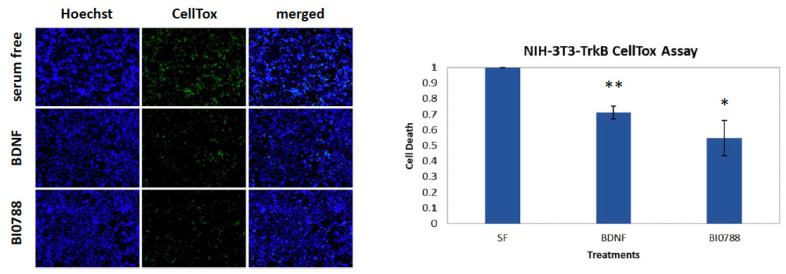
CellTox (cell toxicity) assay for the extract BI0788 in the NIH-3T3-TrkB-expressing cell line. Quantification and representative images of NIH-3T3-TrkB cells untreated and treated with BDNF (500 ng/mL) or extract BI0788 (1 μg/mL) for 24 h after 24 h of serum starvation. *n* = 5 independent experiments; error bars represent SEM; * *p* < 0.05, ** *p* < 0.01.

Initially, a panel of extracts exhibiting interesting chemical profiles among a high number of bacterial strains isolated from East Mediterranean marine sediments and macroorganisms were evaluated using NIH-3T3 cells that are stably transfected and express the functional TrkB neurotrophin receptor (NIH-3T3-TrkB) for their ability to reduce serum deprivation-induced apoptosis in comparison to BDNF, the native ligand of TrkB receptor. Cell death was caused by serum deprivation for 24 h and subsequently BDNF or the different bacterial extracts were added to the media for 24 h to assess (using CellTox kit) whether they were able to reduce the levels of cell death. Among them, the organic extract of *Streptomyces* sp. BI0788 was the most potent, decreasing cell death levels to half as compared to those of the control (serum free) (Figure 2).

Subsequently, the organic extract of strain BI0788, isolated from a marine sediment collected in the Ionian sea and tentatively identified as *Streptomyces* sp., underwent several chromatographic fractionations to yield, in pure form, four new natural products, namely (2*R*,3*R*,4*S*)-2-ethyl-3-hydroxy-4-methylbutan-1,4-olide (**1**), (4*S*,10*R*)-11-oxo-10-methyldodecan-1,4-olide (**4**), (4*S*,10*R*)-11-hydroxy-10-methyldodecan-1,4-olide (**5**), and (4*S*)-10-hydroxy-10-methyldodecan-1,4-olide (**6**), and ten previously reported metabolites (**2**, **3** and **7**–**14**).

**Figure 3:**
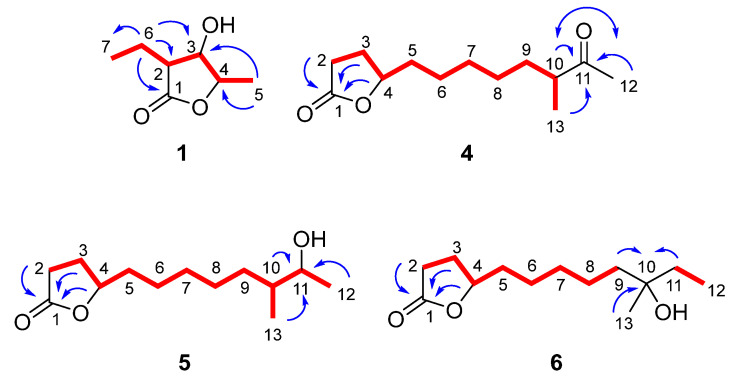
COSY (red bonds) and important HMBC (blue arrows) correlations observed for compounds **1** and **4**–**6**.

Compound **1**, obtained as an amorphous white powder, displayed the molecular formula C_7_H_12_O_3_, as deduced from high-resolution electrospray ionization mass spectrometry (HR-ESIMS) measurements where a sodium adduct ion peak [M + Na]^+^ at *m/z* 167.0679 was detected. The ^1^H and ^13^C NMR data of compound **1** (Table 1, Supplementary Information) revealed the presence of two methyls, one triplet at *δ*_C_ 11.3 and *δ*_H_ 1.07 (CH_3_-7) and one doublet at *δ*_C_ 18.3 and *δ*_H_ 1.44 (CH_3_-5), one methylene at *δ*_C_ 21.3 and *δ*_H_ 1.88/1.68 (CH_2_-6), three methines resonating at *δ*_C_ 49.9 and *δ*_H_ 2.50 (CH-2), *δ*_C_ 78.6 and *δ*_H_ 3.84 (CH-3), and *δ*_C_ 79.7 and *δ*_H_ 4.18 (CH-4), and one quaternary carbon, corresponding to a carbonyl at *δ*_C_ 175.6 (C-1). The planar structure of **1** was determined on the basis of the homonuclear and heteronuclear correlations observed in the HSQC-DEPT, HMBC and COSY spectra (Figure 3). Specifically, a single spin system encompassing all the protonated carbons of the molecule was identified on the basis of the COSY cross-peaks, while the placement of the doublet methyl at C-4 and the ethyl chain at C-2 was further verified from the HMBC correlations of H_3_-5 (*δ*_H_ 1.44) with C-3 (*δ*_C_ 78.6) and C-4 (*δ*_C_ 79.7), and of H_2_-6 (*δ*_H_ 1.88/1.68) with C-1, C-2, C-3, and C-7 (*δ*_C_ 175.6, 49.9, 78.6, and 11.3, respectively). Comparison of the spectroscopic characteristics of compound **1** with those of the co-occurring metabolites **2** and **3**, in combination with the correlations of H-2/H-4 and H-3/H_3_-5 observed in 1D NOE experiments, allowed for the determination of the relative configuration of the chiral centers at C-2, C-3 and C-4. Specifically, the chemical shifts of H-2, H-3, H-4 and H_3_-5, as well as of the corresponding carbons C-2, C-3, C-4 and C-5 of **1**, were similar to those of **2** and **3**, while differing significantly from those of other synthetic analogues featuring the 3-hydroxy-γ-butyrolactone system [14,15,16]. In addition, comparison of the specific optical rotation of **1** with those of **2** and **3** that have the same chiral centers, and for which the absolute configurations have been previously determined, enabled the assignment of the absolute configurations of the asymmetric centers of **1** as 2*R*,3*R*,4*S* [16,17,18]. Thus, compound **1** was identified as a new natural product, which was designated as (2*R*,3*R*,4*S*)-2-ethyl-3-hydroxy-4-methylbutan-1,4-olide.

Compound **4** was obtained as a yellow oil. The HR-ESIMS measurements showed a sodium adduct ion peak [M + Na]^+^ at *m/z* 249.1456, indicating the molecular formula C_13_H_22_O_3_, which in turn suggested that **4** had three degrees of unsaturation. The planar structure of compound **4** was assigned by interpretation of its 1D and 2D NMR spectroscopic data. In particular, the ^1^H and ^13^C NMR spectroscopic data of compound **4** (Table 2) showed the presence of thirteen carbons, including one singlet methyl at *δ*_C_ 28.0 and *δ*_H_ 2.11 (CH_3_-12), one doublet methyl at *δ*_C_ 16.3 and *δ*_H_ 1.06 (CH_3_-13), seven methylenes, one oxygenated methine at *δ*_C_ 83.3 and *δ*_H_ 4.45 (CH-4), one deshielded methine at *δ*_C_ 47.1 and *δ*_H_ 2.48 (CH-10), and two quaternary carbons at *δ*_C_ 177.2 (C-1) and 212.8 (C-11), corresponding to one ester and one ketone carbonyl, respectively, with the latter two accounting for two of the three degrees of unsaturation, indicating the monocyclic structure of compound **4**. The correlations observed in the COSY spectrum revealed the presence of a single spin system, while the HMBC correlations of H_2_-2, H_2_-3 and H-4 with C-1 supported the presence of a γ-lactone. Additionally, the HMBC correlations of H-10, H_3_-12 and H_3_-13 with C-11 connected C-10 with an acetyl group (*δ*_C_ 212.8 and *δ*_C_ 28.0/*δ*_H_ 2.11) and concluded the structure of **4** (Figure 3). Comparison of the ^1^H NMR data of **4** with those of **7** suggested that compound **4** was the saturated analogue of butenolide **7**. The spectroscopic and physical characteristics of (4*S*,10*R*)-11-oxo-10-methyldodecan-1,4-olide (**4**), described here for the first time as a natural product, are identical to those reported for a semisynthetic derivative of compound **7** [19].

Based on the analysis of the spectroscopic data of **5** and **6** (Table 2, Figure 3), it was obvious that compound **5** was the saturated analogue of either **8** or **9**, whereas **6** was the saturated analogue of **10**, as also indicated by the HR-ESIMS measurements of **5** and **6**, suggesting the molecular formula C_13_H_24_O_3_ for both. Hydrogenation of Δ^2^ and subsequent reduction of the ketone carbonyl of **7** afforded the two epimeric alcohols at C-11, among which one was identical to compound **5**. In a similar manner, hydrogenation of the lactone ring of **10** yielded compound **6**, proving in both cases that **5** and **6** were the saturated analogues of **8** and **10**, respectively, and determining the absolute configurations at the chiral centers.

Compounds **2**, **3** and **7**–**14** were identified as (−)-blastmycinolactol (**2**) [16,17,18,20], (−)-8-methylblastmycinolactol (**3**) [16], (4*S*,10*R*)-11-oxo-10-methyldodec-2-en-1,4-olide (**7**) [16,19,21], (4*S*,10*R*,11*S*)-11-hydroxy-10-methyldodec-2-en-1,4-olide (**8**) [19], (4*S*,10*R*,11*R*)-11-hydroxy-10-methyldodec-2-en-1,4-olide (**9**) [19], (4*S*)-10-hydroxy-10-methyldodec-2-en-1,4-olide (**10**) [19,21], (4*S*)-10-hydroxy-11-oxo-10-methyldodec-2-en-1,4-olide (**11**) [21], (4*S*)-10,11-dihydroxy-10-methyldodec-2-en-1,4-olide (**12**) [22], (4*S*,10*R*)-10-methyldodec-2-en-1,4-olide (**13**) [19] and (4*S*)-10-hydroxy-10-methylundec-2-en-1,4-olide (**14**) [16] by comparison of their spectroscopic data and physical characteristics with those reported in the literature.

**Figure 4:**
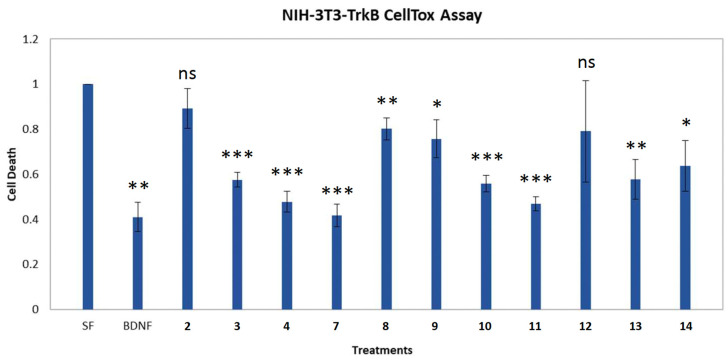
CellTox (cell toxicity) assay for compounds **2**–**4** and **7**–**14** in the NIH-3T3-TrkB cell line. Quantification of cells untreated and treated with BDNF (500 ng/mL) or the compounds (1 μM) for 24 h after 24 h of serum starvation. *n* = 3 independent experiments; error bars represent SEM; * *p* < 0.05, ** *p* < 0.01, *** *p* < 0.001; ns: not significant.

Following the isolation of compounds **1**–**14**, we proceeded with the evaluation of the protective activity of compounds **2**–**4** and **7**–**14**, which were isolated in sufficient amounts on NIH-3T3-TrkB cells. Compounds **3**, **4**, **7**–**11**, **13** and **14** were able to significantly reduce cell death at the concentration of 1 μM (Figure 4).

**Figure 5:**
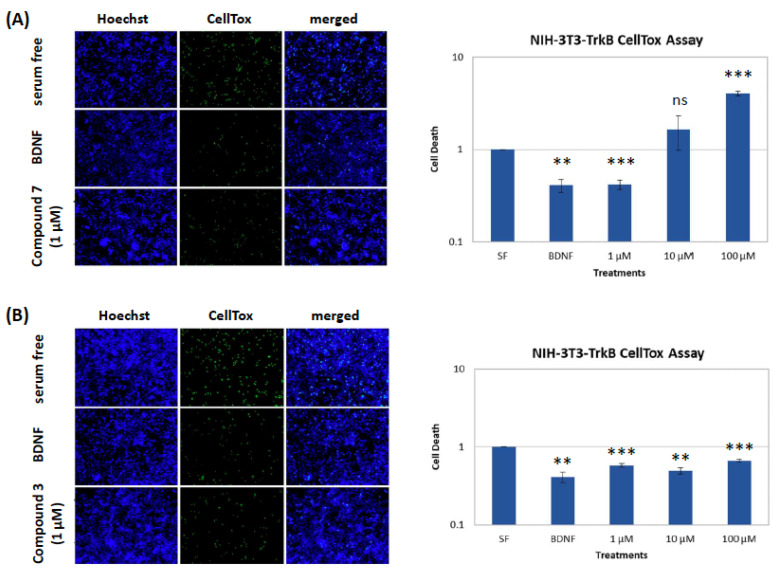
CellTox (cell toxicity) assay for compounds **3** and **7** at different concentrations in the NIH-3T3-TrkB-expressing cell line. (**A**). Quantification and representative images of NIH-3T3-TrkB cells untreated and treated with BDNF (500 ng/mL) or compound **7** (1, 10 and 100 μM) for 24 h after 24 h of serum starvation. (**B**). Quantification and representative images of NIH-3T3-TrkB cells untreated and treated with BDNF (500 ng/mL) or compound **3** (1, 10 and 100 μM) for 24 h after 24 h of serum starvation. *n* = 3 independent experiments; error bars represent SEM; ** *p* < 0.01, *** *p* < 0.001; ns: not significant.

Compound **7** showed the greatest reduction at 1 μM, similar to BDNF levels, but this effect was not reproduced at higher concentrations of **7** (10 and 100 μM) with cell death levels dramatically increased at higher concentrations as compared to those of the serum free control (Figure 5A). In contrast, compound **3** was able to decrease cell death levels not only at 1 μM, but also at 10 and 100 μM (Figure 5B).

**Figure 6:**
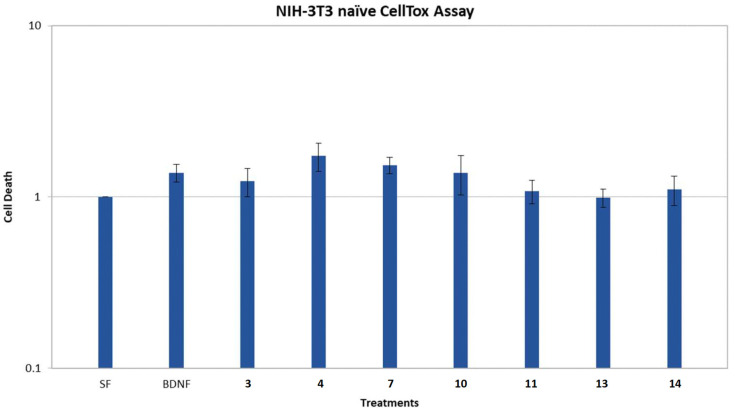
CellTox (cell toxicity) assay for compounds **3**, **4**, **7**, **10**, **11**, **13** and **14** in the NIH-3T3 naïve cell line. Quantification of cells untreated and treated with BDNF (500 ng/mL) or the compounds (1 μM) for 24 h after 24 h of serum starvation. *n* = 2 independent experiments; error bars represent SEM.

To confirm that the effect of the isolated compounds on cell death levels is indeed mediated via the TrkB receptor, their activity was also evaluated on NIH-3T3 naïve cells, which are not transfected to express the TrkB receptor. As shown in Figure 6, the effect on cell death reduction after treatment with BDNF or the compounds was abolished in the absence of the TrkB receptor, clearly indicating that the TrkB receptor is necessary for the protective activity of all active compounds.

**Figure 7:**
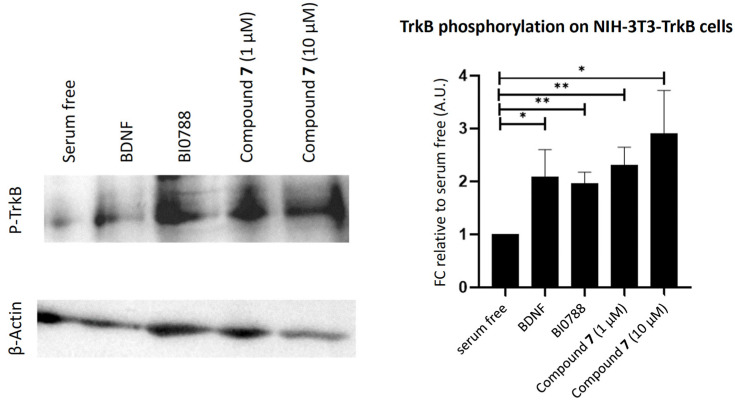
Phosphorylation assay for extract BI0788 and compound **7** on NIH-3T3-TrkB cell line. Quantification and representative images (phospho-TrkB and β-Actin) of the Western Blot assay on cells untreated and treated with BDNF (500 ng/mL) or extract BI0788 (1 μg/mL) or compound **7** (1 and 10 μM) for 20 min after 6 h of serum starvation. *n* = 3–4 independent experiments; error bars represent SEM. * *p* < 0.05, ** *p* < 0.01.

In order to further decipher the mechanism of action of extract BI0788 and compound **7** as one of the most promising compounds, we proceeded with TrkB activity experiments. Using Western Blot analysis, we depicted their ability to phosphorylate TrkB in NIH-3T3-Trk-expressing cells (Figure 7). The results manifest the rapid activation properties of both the extract BI0788 and compound **7** on the TrkB receptor, highlighting a new structural substrate for developing novel micro-molecular agonists of this neurotrophin receptor.

## 3. Discussion

The organic extract of the marine-derived bacterial strain BI0788 showed an ability to significantly reduce cell death in a TrkB-dependent cell population. Several compounds isolated from this extract showed equal ability to decrease cell death on the same cell population at 1 μM concentration. Among them, compound **7** presented the most powerful effect on reducing cell death levels at 1 μM, in comparison to the remaining compounds, reaching levels comparable to BDNF. Interestingly, the cytoprotective ability of compound **7** at 1 μΜ was not preserved at higher concentrations since it displayed higher levels of cell death at 10 and 100 μM as compared to those of the serum free control. Therefore, compound **7** may function variably, possibly as a result of its small size, unlike the native neurotrophin ligand BDNF, acting as either an agonist or an antagonist, depending on its concentration. Small molecules that have been developed so far as direct or indirect agonists or antagonists of neurotrophin receptors are known to bind in different sites of the receptor in comparison to neurotrophic factors of bigger size [23]. The dual activity of compound **7** could be attributed to different binding sites on the TrkB receptor, depending on its concentration. Interestingly, another promising candidate, compound **3**, reduced cell death levels at all concentrations (1, 10 and 100 μM) as compared to the serum free control, indicating that small structural changes could reflect significant functional differences on neurotrophin receptor signaling properties.

To examine if the effects of the compounds on cell survival were selectively dependent on the TrkB receptor function, NIH-3T3 cells that do not express TrkB were used. Neither BDNF nor the compounds reversed cell death levels of the serum free control on NIH-3T3 naïve cells, providing strong evidence that the activity of the particular compounds is exclusively mediated by TrkB, either directly or through the trans-activation of the neurotrophin receptor.

In order to decipher the direct effects of active compounds on TrkB activation, we performed experiments to quantitatively measure TrkB phosphorylation, which represents the first step on the receptor’s activation. Conjointly, a 20 min incubation with extract BI0788 or compound **7** was able to significantly phosphorylate the TrkB receptor on NIH-3T3-TrkB-expressing cells. This rapid action, even if it does not prove their direct interaction with the TrkB receptor, clearly depicts a significant agonistic effect.

## 4. Materials and Methods

### 4.1. General Experimental Procedures

Optical rotations were measured on a Krüss model P3000 polarimeter (A. KRÜSS Optronic GmbH, Hamburg, Germany) with a 0.5 dm cell. UV spectra were obtained on a Perkin Elmer Lambda 40 spectrophotometer (PerkinElmer Ltd., Buckinghamshire, UK). IR spectra were obtained on a Bruker Alpha II spectrometer (Bruker Optik GmbH, Ettlingen, Germany). 1D and 2D NMR spectra were recorded on a Bruker DRX 400 (Bruker BioSpin GmbH, Rheinstetten, Germany) spectrometer, using standard Bruker pulse sequences at room temperature. Chemical shifts are reported on the *δ* (ppm) scale using TMS as internal standard. High-resolution electrospray ionization (ESI) mass spectra were measured on a Thermo Scientific LTQ Orbitrap Velos mass spectrometer (Thermo Fisher Scientific, Bremen, Germany). Low-resolution electron ionization (EI) mass spectra were measured on a Hewlett–Packard 5973 mass spectrometer (Agilent Technologies, Santa Clara, CA, USA) or on a Thermo Electron Corporation DSQ mass spectrometer (Thermo Electron Corporation, Austin, TX, USA). Normal- and reversed-phase column chromatography separations were performed with Kieselgel Si 60 (Merck, Darmstadt, Germany) and Kieselgel RP-18 (Merck, Darmstadt, Germany), respectively. HPLC separations were conducted on (i) a Cecil 1100 Series liquid chromatography pump (Cecil Instruments Ltd., Cambridge, UK) equipped with a GBC LC-1240 refractive index detector (GBC Scientific Equipment, Braeside, VIC, Australia), (ii) an Agilent 1100 liquid chromatography system equipped with a refractive index detector (Agilent Technologies, Waldbronn, Germany), or (iii) a Waters 600 liquid chromatography pump (Waters, Milford, MA, USA) with a Waters 410 refractive index detector (Waters, Milford, MA, USA), using the following columns: (i) Econosphere C_18_ 10u (250 × 10 mm, Grace, Columbia, MD, USA), (ii) Kromasil 100-7-C_18_ (250 × 10 mm, Akzonobel, Eka Chemicals AB, Separation Products, Bohus, Sweden), (iii) Luna C_18_ (2) 100A 10u (250 × 10 mm, Phenomenex, Torrance, CA, USA), (iv) Econosphere Silica 10u (250 × 10 mm, Grace, Columbia, MD, USA), (v) Kromasil 100-10-SIL (250 × 10 mm, Akzonobel, Eka Chemicals AB, Separation Products, Bohus, Sweden), (vi) Supelcosil SPLC-Si 5 µm (250 × 10 mm, Supelco, Bellefonte, PA, USA), or (vii) Chiralcel OD 10 μm (250 × 10 mm, Daicel Chemical Industries Ltd., Osaka, Japan). TLC was performed with Kieselgel 60 F_254_ aluminum-backed plates (Merck, Darmstadt, Germany), and spots were visualized after spraying with 15% (*v*/*v*) H_2_SO_4_ in MeOH reagent and heating at 100 °C for 1 min.

### 4.2. Biological Material

The bacterial strain BI0788 was isolated from a marine sediment collected at Vassiliki bay at the island of Lefkada in the Ionian sea from a depth of 4 m in April 2014. The strain was identified as a *Streptomyces* sp. based on comparison of its 16S ribosomal RNA (rRNA) sequences with data from the Genbank database of the National Center for Biotechnology Information (NCBI) using the Basic Local Alignment Search Tool (BLAST), sharing approx. 98.5% gene sequence identity with its closest neighbor (GenBank accession number KM103736.1). The strain is deposited at the strain collection/microbank of the Section of Pharmacognosy and Chemistry of Natural Products, Department of Pharmacy, National and Kapodistrian University of Athens.

### 4.3. Fermentation, Extraction and Isolation

The bacterial strain was streaked from a glycerol stock onto 20 freshly prepared agar plates containing a seawater-based (A1BFe+C) medium (10 g starch, 4 g yeast extract, 2 g peptone, 1 g CaCO_3_, 0.1 g KBr, and 0.04 g Fe_2_(SO_4_)_3_ 5H_2_O per liter of filtered seawater) (Bugni et al., 2006). After 3 days, when sufficient growth of the bacterial strain was observed, mycelia were picked from the agar plates and were inoculated into 1 L flasks containing 500 mL of the same seawater-based medium, (10% *v*/*v* inoculum), to a total of 10 L of liquid medium, which were incubated at 23 °C for 7 days while shaking at 130 rpm in an orbit shaker.

At the end of the fermentation period, Amberlite XAD-7HP resin (Sigma-Aldrich, St. Louis, MO, USA) (20 g/L) was added to each flask to adsorb extracellular metabolites. The culture and resin were shaken overnight at low speed. The broth was centrifuged, and the pellet (resin and cell mass) was extracted twice for 24 h with Me_2_CO (4 L in total). Filtration of the extract and removal of the solvent under vacuum at 40 °C afforded a solid residue. Additionally, the supernatant was successively partitioned with EtOAc. Evaporation of the solvents under vacuum at 40 °C afforded an additional solid residue, which was combined with the Me_2_CO soluble residue to yield the total extract (16.817 g).

Subsequently, the total extract of BI0788 was subjected to vacuum liquid chromatography on silica gel, using cyclohexane, with increasing amounts of EtOAc, followed by EtOAc, with increasing amounts of MeOH as mobile phase, yielding 11 fractions (788A–788K). Fractions 788B-788D (5–30% EtOAc in cyclohexane, 555.9 mg) were combined and further fractionated by vacuum liquid chromatography, using *n*-pentane with increasing amounts of Et_2_O as mobile phase, resulting in 8 fractions (788B1-788B8). Fraction 788B6 (30–50% Et_2_O in *n*-pentane, 28.9 mg) was purified by normal-phase HPLC, using cyclohexane/EtOAc (88:12) as eluent to afford **13** (2.2 mg). Fraction F (45% EtOAc in cyclohexane, 186.6 mg) was further fractionated by solid phase extraction, using water with increasing amounts of MeOH to yield 10 fractions (788F1-788F10). Fractions 788F1 and 788F2 (20–30% MeOH in H_2_O, 10.6 mg) were combined and further purified by reversed-phase HPLC, using MeOH/H_2_O (60:40) as eluent to afford **2** (1.1 mg) and **3** (1.2 mg). Fractions 788G (50–60% EtOAc in cyclohexane, 136.3 mg) and 788H (80% EtOAc in cyclohexane to 100% EtOAc, 80.3 mg) were further fractionated by gravity column chromatography on silica gel, using cyclohexane with increasing amounts of EtOAc as the mobile phase to yield 10 fractions, respectively (788G1-788G10, and 788H1-H10). Fractions 788G4 (35% EtOAc in cyclohexane, 27.2 mg) and 788G5 (35% EtOAc in cyclohexane, 23.8 mg) were purified by normal-phase HPLC, using cyclohexane/EtOAc (65:35) and toluene/EtOAc (75:25) as eluent to afford **1** (0.6 mg), **4** (1.4 mg) and **7** (16.8 mg). Fraction 788H7 (50–65% EtOAc in cyclohexane, 35.1 mg) was subjected to a first round of purification on normal-phase HPLC, using cyclohexane/Me_2_CO (65:35) as eluent, then through sequential purification on chiral-phase HPLC using a mixture of *n*-hexane with decreasing amounts of *iso*-propanol (94:6 to 97:3) as eluent, yielding **5** (0.6 mg), **6** (0.4 mg), **8** (2.0 mg), **9** (1.6 mg), **10** (7.5 mg), and **11** (0.8 mg). Fractions 788I (5–10% MeOH in EtOAc, 55.7 mg) and 788J (20% MeOH in EtOAc, 72.5 mg) were further fractionated by gravity column chromatography on silica gel, using cyclohexane with increasing amounts of Me_2_CO as the mobile phase to yield 6 fractions and 11 fractions, respectively (788I1-788I6, and 788J1-J11). Fractions 788I2 (30% Me_2_CO in cyclohexane, 24.9 mg) and 788J6 (35% Me_2_CO in cyclohexane, 12.2 mg) were sequentially purified by normal-phase HPLC, using cyclohexane/Me_2_CO (65:35 and 60:40) as eluent to afford **12** (1.1 mg), and **14** (2.0 mg).

(2*R*,3*R*,4*S*)-2-Ethyl-3-hydroxy-4-methylbutan-1,4-olide (**1**): white solid; [α]${}_{D}^{25}$ −8.2 (*c* 0.05, MeOH); UV (MeOH) *λ*_max_ (log *ε*) 200.5 (2.82) nm; IR (thin film) *v*_max_ 3450, 2980, 2920, 2880, 1750, 1460, 1380, 1180, 1050 cm^−1^; ^1^H and ^13^C NMR data, see Table 1; HR-ESIMS *m/z* 167.0679 [M + Na]^+^ (calcd. for C_7_H_12_O_3_Na, 167.0684).

(−)-Blastmycinolactol (**2**): white solid; [α]${}_{D}^{25}$ −9.7 (*c* 0.10, MeOH); ^1^H NMR, see Table 1; EIMS *m*/*z* 172 [M]^+^.

(−)-8-Methylblastmycinolactol (**3**): white solid; [α]${}_{D}^{25}$ −12.3 (*c* 0.10, MeOH); ^1^H NMR, see Table 1; EIMS *m*/*z* 186 [M]^+^.

(4*S*,10*R*)-11-Oxo-10-methyldodecan-1,4-olide (**4**): yellow oil; [α]${}_{D}^{25}$ −21.8 (*c* 0.09, MeOH); UV (MeOH) *λ*_max_ (log *ε*) 200.5 (3.23), 215 (3.04) nm; IR (thin film) *v*_max_ 2930, 2860, 1770, 1710, 1460, 1350, 1180 cm^−1^; ^1^H and ^13^C NMR data, see Table 2; HR-ESIMS *m/z* 249.1456 [M + Na]^+^ (calcd. for C_13_H_22_O_3_Na, 249.1467).

(4*S*,10*R*)-11-Hydroxy-10-methyldodecan-1,4-olide (**5**): yellow oil; [α]${}_{D}^{25}$ −12.0 (*c* 0.17, MeOH); UV (MeOH) *λ*_max_ (log *ε*) 200.5 (3.21), 221 (2.84) nm; IR (thin film) *v*_max_ 3450, 2920, 2850, 1740, 1460, 1380, 1180 cm^−1^; ^1^H and ^13^C NMR data, see Table 2; HR-ESIMS *m/z* 251.1614 [M + Na]^+^ (calcd. for C_13_H_24_O_3_Na, 251.1623).

(4*S*)-10-Hydroxy-10-methyldodecan-1,4-olide (**6**): yellow oil; [α]${}_{D}^{25}$ −22.3 (*c* 0.54, MeOH); UV (MeOH) *λ*_max_ (log *ε*) 201 (3.63), 219 (3.52) nm; IR (thin film) *v*_max_ 3450, 2920, 2850, 1740, 1460, 1380, 1180 cm^−1^; ^1^H and ^13^C NMR data, see Table 2; HR-ESIMS *m/z* 251.1617 [M + Na]^+^ (calcd. for C_13_H_24_O_3_Na, 251.1623).

(4*S*,10*R*)-11-Oxo-10-methyldodec-2-en-1,4-olide (**7**): yellow oil; [α]${}_{D}^{25}$ +42.4 (*c* 0.20, MeOH); ^1^H NMR, see [16,19,21]; EIMS *m*/*z* 224 [M]^+^.

(4*S*,10*R*,11*S*)-11-Hydroxy-10-methyldodec-2-en-1,4-olide (**8**): yellow oil; [α]${}_{D}^{25}$ +58.3 (*c* 0.10, MeOH); ^1^H NMR, see [19]; EIMS *m*/*z* 226 [M]^+^.

(4*S*,10*R*,11*R*)-11-Hydroxy-10-methyldodec-2-en-1,4-olide (**9**): yellow oil; [α]${}_{D}^{25}$ +37.7 (*c* 0.10, MeOH); ^1^H NMR, see [19]; EIMS *m*/*z* 226 [M]^+^.

(4*S*)-10-Hydroxy-10-methyldodec-2-en-1,4-olide (**10**): yellow oil; [α]${}_{D}^{25}$ +62.8 (*c* 0.13, MeOH); ^1^H NMR, see [19,21]; EIMS *m*/*z* 226 [M]^+^.

(4*S*)-10-Hydroxy-11-oxo-10-methyldodec-2-en-1,4-olide (**11**): yellow oil; [α]${}_{D}^{25}$ +20.1 (*c* 0.10, MeOH); ^1^H NMR, see [21]; EIMS *m*/*z* 240 [M]^+^.

(4*S*)-10,11-Dihydroxy-10-methyldodec-2-en-1,4-olide (**12**): yellow oil; [α]${}_{D}^{25}$ +20.3 (*c* 0.10, MeOH); ^1^H NMR, see [22]; EIMS *m*/*z* 242 [M]^+^.

(4*S*,10*R*)-10-Methyldodec-2-en-1,4-olide (**13**): yellow oil; [α]${}_{D}^{25}$ +48.2 (*c* 0.10, MeOH); ^1^H NMR, see [19]; EIMS *m*/*z* 210 [M]^+^.

(4*S*)-10-Hydroxy-10-methylundec-2-en-1,4-olide (**14**): yellow oil; [α]${}_{D}^{25}$ +18.7 (*c* 0.10, MeOH); ^1^H NMR, see [16]; EIMS *m*/*z* 212 [M]^+^.

### 4.4. Preparation of Compound **5**

To a solution of **7** (10.0 mg) in EtOAc (4 mL), Pd/C (1 mg) was added and the resulting mixture was stirred under H_2_ atmosphere overnight. Then, it was passed through a celite pad and the filtrate was evaporated to dryness. To an ice-cold solution of the hydrogenated intermediate (8.5 mg) in MeOH (0.1 mL), a solution of NaBH_4_ (2.2 mg, 0.057 mmol, 1.5 eq) in MeOH (0.3 mL) was added dropwise. The reaction mixture was left at RT for 1 h (monitored with TLC and ESIMS). It was quenched with 3 drops of water and evaporated to dryness, affording a mixture of the two epimeric alcohols at C-11. Consecutive purifications on chiral-phase HPLC, using a mixture of *n*-hexane/*iso*-propanol (94:6 and 97:3) as eluent, afforded **5** in pure form (3.0 mg).

### 4.5. Preparation of Compound **6**

To a solution of **10** (6.0 mg) in EtOAc (3 mL), Pd/C (1 mg) was added and the resulting mixture was stirred under H_2_ atmosphere overnight. Then, it was passed through a celite pad and the filtrate was evaporated to dryness. Consecutive purifications on chiral-phase HPLC, using a mixture of n-hexane/iso-propanol (94:6 and 97:3) as eluent, afforded **6** (3.5 mg).

### 4.6. TrkB Activation

Stably transfected NIH-3T3-TrkB-expressing cells were used to test the ability of the extracts or compounds to activate the TrkB receptor under serum deprivation conditions. Cultures were carried out in High-Glucose DMEM medium supplemented with 10% fetal bovine serum (FBS), 100 units/mL penicillin and 100 µg/mL streptomycin in a humidified 5% CO_2_ atmosphere at 37 °C.

### 4.7. CellTox Green Cytotoxicity Assay

Naïve NIH-3T3 or NIH-3T3-TrkB-expressing cells plated in 96-well plates at 10,000 cells/well were serum starved for 24 h. Compound treatments were performed at 1–100 μM for 24 h, while BDNF was used as a positive control at 500 ng/mL. For dead and total cell counting, CellTox™ Green Dye (2000×, Promega, Tokyo, Japan) and Hoechst 33342 Solution (10,000×), respectively, were used. For image capture, a ZEISS Axio Vert.A1 microscope and the ImageJ software (https://imagej.nih.gov/ij/, accessed on 30 April 2023) was used.

### 4.8. Western Blot

NIH-3T3-TrkB-expressing cells were plated in 12-well plates at 100,000 cells/well and the following day were serum deprived for 6 h. Compound treatments to assess TrkB activation were performed at 1 μM for 20 min, while BDNF was used as a positive control at 500 ng/mL. Lysis was performed using a lysis buffer on ice (Thermo Scientific Pierce IP Lysis Buffer) with a Millipore phospho-protease inhibitor cocktail by Millipore for 10 min. After adding the loading buffer (5× Laemni) to 25 μg of the protein sample, samples were incubated at 95 °C for 5 min and subjected to SDS-PAGE. Nitrocellulose membrane transfer was performed at 350 mA for 2 h, followed by blocking at room temperature with 5% bovine serum albumin (BSA) for 1 h and primary antibody incubation in blocking solutions at 4 °C overnight and detection with HRP-conjugated secondary antibodies. ECL solution was used to detect chemiluminescence. Primary antibodies used are Phospho-TrkB (Tyr816) Millipore # ABN1381 and Anti-TrkB Abcam #ab33655, β-Actin.

## 5. Conclusions

The chemical investigation of the organic extract of *Streptomyces* sp. BI0788, an actinobacterial strain isolated from a marine sediment collected from the Ionian sea that showed an ability to significantly reduce cell death in a TrkB-dependent cell population, led to the isolation and structure elucidation of four new butanolides (**1** and **4**–**6**), along with two previously reported butanolides (**2** and **3**) and eight previously reported butenolides (**7**–**14**). Among compounds **2**–**4** and **7**–**14** that were isolated in sufficient amounts and evaluated for their activity on NIH-3T3-TrkB cells, compound **7** showed the greatest reduction at 1 μM, similar to the levels of the native neurotrophin ligand BDNF.

This study demonstrates for the first time the action of natural small molecules that have the ability to activate the TrkB receptor and promote cell survival, one of the most important functions mediated by the receptor. Over the last years, many research efforts have focused on developing BDNF mimetics with suitable pharmacological properties in order to be used as new therapeutics against neurodegenerative diseases. Hence, our results suggest that these γ-lactone-containing compounds could have great potential in various neurological disorders as lead molecules for further investigations in relevant mouse models and eventually in human clinical trials.

## Figures and Tables

**Table 1:** ^13^C and ^1^H NMR data (*δ* in ppm, *J* in Hz) of compounds **1**–**3** in CDCl_3_.

Position	1		2	3
	*δ*_C_ ^†^	*δ*_H_	*δ*_H_	*δ*_H_
1	175.6			
2	49.9	2.50 ddd (8.4, 7.4, 5.8)	2.53 ddd (8.6, 7.5, 5.7)	2.51 m
3	78.6	3.84 dd (8.4, 7.0)	3.83 m	3.82 m
4	79.7	4.18 dq (7.0, 6.3)	4.18 dq (6.9, 6.3)	4.17 dq (6.9, 6.3)
5	18.3	1.44 d (6.3)	1.44 d (6.3)	1.44 d (6.3)
6	21.3	1.88 dqd (7.5, 7.5, 5.8), 1.68 dqd (7.5, 7.5, 7.4)	1.86 m, 1.59 m	1.84 m, 1.58 m
7	11.3	1.07 t (7.5)	1.47 m	1.37 m, 1.31 m
8			1.36 m	1.56 m
9			0.91 t (7.2)	0.90 d (6.5)
10				0.90 d (6.5)

^† 13^C chemical shifts were determined through HMBC correlations.

**Table 2:** ^13^C and ^1^H NMR data (*δ* in ppm, *J* in Hz) of compounds **4**–**6** in CDCl_3_.

Position	4		5		6	
	*δ*_C_ ^†^	*δ*_H_	*δ*_C_ ^†^	*δ*_H_	*δ*_C_ ^†^	*δ*_H_
1	177.2		177.3		177.3	
2	28.5	2.51 m	28.3	2.511 dd (9.6, 6.4), 2.509 dd (9.4, 7.4)	28.5	2.51 m
3	27.8	2.30 m, 1.83 dddd (12.7, 9.6, 9.4, 8.1)	27.9	2.30 m, 1.83 dddd (12.7, 9.6, 9.4, 8.0)	27.8	2.30 m, 1.83 dddd (12.7, 9.6, 9.4, 8.1)
4	83.3	4.45 dddd (8.1, 7.7, 7.7, 5.4)	80.8	4.45 dddd (8.0, 7.7, 7.7, 5.4)	80.8	4.45 dddd (8.1, 7.7, 7.7, 5.4)
5	35.4	1.72 m, 1.59 m	35.5	1.72 m, 1.59 m	35.4	1.72 m, 1.59 m
6	25.2	1.46 m, 1.39 m	25.2	1.46 m, 1.37 m	25.0	1.47 m, 1.39 m
7	29.3	1.33 m, 1.24 m	29.5	1.33 m, 1.25 m	29.4	1.33 m, 1.23 m
8	27.0	1.38 m, 1.25 m	27.0	1.38 m, 1.23 m	23.3	1.38 m, 1.32 m
9	32.6	1.64 m, 1.32 m	32.3	1.43 m, 1.07 m	40.8	1.41 m
10	47.1	2.48 m	39.9	1.46 m	72.8	
11	212.8		71.6	3.63 dq (6.3, 6.3)	33.9	1.46 q (7.5)
12	28.0	2.11 s	19.4	1.11 d (6.3)	8.1	0.87 t (7.5)
13	16.3	1.06 d (7.0)	14.6	0.85 d (6.8)	26.2	1.12 s

^† 13^C chemical shifts were determined through HMBC correlations.

## Data Availability

The data presented in this study are available in the present article and the Supplementary Material.

## Supplementary Materials

The following supporting information can be downloaded at: https://www.mdpi.com/article/10.3390/md21090465/s1, Figures S1–S42: 1D and 2D NMR and MS spectra of compounds **1**–**14**.
