# Supplementary material for: Butanolides and Butenolides from a Marine-Derived Streptomyces sp. Exert Neuroprotective Activity through Activation of the TrkB Neurotrophin Receptor
Source: Mar Drugs. 2023 Aug 25;21(9):465. doi: 10.3390/md21090465 (PMC10532803; doi:10.3390/md21090465)
Supplement: Supplementary file 1 [file marinedrugs-21-00465-s001.zip › marinedrugs-2552877-supplementary.pdf]

Supplementary Materials for

Butanolides and Butenolides from a Marine-derived *Streptomyces* sp. Exert Neuroprotective Activity through Activation of the TrkB Neurotrophin Receptor

Paolo Giaccio [1], Despoina Charou [2,3], Dafni-Ioanna Diakaki [1], Anna Chita [2,3], Achille Gravanis [2,3], Ioannis Charalampopoulos [2,3], Vassilios Roussis [1] and Efstathia Ioannou [1,*]

[1] Section of Pharmacognosy and Chemistry of Natural Products, Department of Pharmacy, National and Kapodistrian University of Athens, Panepistimiopolis Zografou, Athens 15771, Greece; pgiaccio@pharm.uoa.gr (P.G.); dafnid@pharm.uoa.gr (D.-I.D.); roussis@pharm.uoa.gr (V.R.)

[2] Department of Pharmacology, Medical School, University of Crete, Heraklion 71003, Greece; dcharou@gmail.com (D.C.); anna.chita2@gmail.com (A.C.); gravanis@med.uoc.gr (A.G.); charalampn@uoc.gr (I.C.)

[3] Institute of Molecular Biology & Biotechnology, Foundation for Research & Technology-Hellas (IMBB-FORTH), Heraklion 70013, Greece

* Correspondence: eioannou@pharm.uoa.gr; Tel.: +30 210 727 4913

Table of Contents

**Figure S1.**	^{1}H NMR spectrum ($CDCl_3$) of compound **1**.	S3
**Figure S2.**	HSQC-DEPT spectrum ($CDCl_3$) of compound **1**.	S3
**Figure S3.**	HMBC spectrum ($CDCl_3$) of compound **1**.	S4
**Figure S4.**	COSY spectrum ($CDCl_3$) of compound **1**.	S4
**Figure S5.**	1D NOE difference spectrum ($CDCl_3$) of compound **1** upon irradiation of H-2.	S5
**Figure S6.**	1D NOE difference spectrum ($CDCl_3$) of compound **1** upon irradiation of H-3.	S5
**Figure S7.**	HR-ESIMS spectrum of compound **1**.	S6
**Figure S8.**	^{1}H NMR spectrum ($CDCl_3$) of compound **2**.	S7
**Figure S9.**	EIMS spectrum of compound 2.	S7
**Figure S10.**	^{1}H NMR spectrum ($CDCl_3$) of compound **3**.	S8
**Figure S11.**	EIMS spectrum of compound **3**.	S8
**Figure S12.**	^{1}H NMR spectrum ($CDCl_3$) of compound **4**.	S9
**Figure S13.**	HSQC-DEPT spectrum ($CDCl_3$) of compound **4**.	S9
**Figure S14.**	HMBC spectrum ($CDCl_3$) of compound **4**.	S10
**Figure S15.**	COSY spectrum ($CDCl_3$) of compound **4**.	S10
**Figure S16.**	HR-ESIMS spectrum of compound **4**.	S11
**Figure S17.**	^{1}H NMR spectrum ($CDCl_3$) of compound **5**.	S12
**Figure S18.**	HSQC-DEPT spectrum ($CDCl_3$) of compound **5**.	S12
**Figure S19.**	HMBC spectrum ($CDCl_3$) of compound **5**.	S13
**Figure S20.**	COSY spectrum ($CDCl_3$) of compound **5**.	S13
**Figure S21.**	HR-ESIMS spectrum of compound **5**.	S14
**Figure S22.**	^{1}H NMR spectrum ($CDCl_3$) of compound **6**.	S15
**Figure S23.**	HSQC-DEPT spectrum ($CDCl_3$) of compound **6**.	S15
**Figure S24.**	HMBC spectrum ($CDCl_3$) of compound **6**.	S16
**Figure S25.**	COSY spectrum ($CDCl_3$) of compound **6**.	S16
**Figure S26.**	HR-ESIMS spectrum of compound **6**.	S17
**Figure S27.**	^{1}H NMR spectrum ($CDCl_3$) of compound **7**.	S18
**Figure S28.**	EIMS spectrum of compound **7**.	S18
**Figure S29.**	^{1}H NMR spectrum ($CDCl_3$) of compound **8**.	S19
**Figure S30.**	EIMS spectrum of compound **8**.	S19
**Figure S31.**	^{1}H NMR spectrum ($CDCl_3$) of compound **9**.	S20
**Figure S32.**	EIMS spectrum of compound **9**.	S20
**Figure S33.**	^{1}H NMR spectrum ($CDCl_3$) of compound **10**.	S21
**Figure S34.**	EIMS spectrum of compound **10**.	S21
**Figure S35.**	^{1}H NMR spectrum ($CDCl_3$) of compound **11**.	S22
**Figure S36.**	EIMS spectrum of compound **11**.	S22
**Figure S37.**	^{1}H NMR spectrum ($CDCl_3$) of compound **12**.	S23
**Figure S38.**	EIMS spectrum of compound **12**.	S23
**Figure S39.**	^{1}H NMR spectrum ($CDCl_3$) of compound **13**.	S24
**Figure S40.**	EIMS spectrum of compound **13**.	S24
**Figure S41.**	^{1}H NMR spectrum ($CDCl_3$) of compound **14**.	S25
**Figure S42.**	EIMS spectrum of compound **14**.	S25

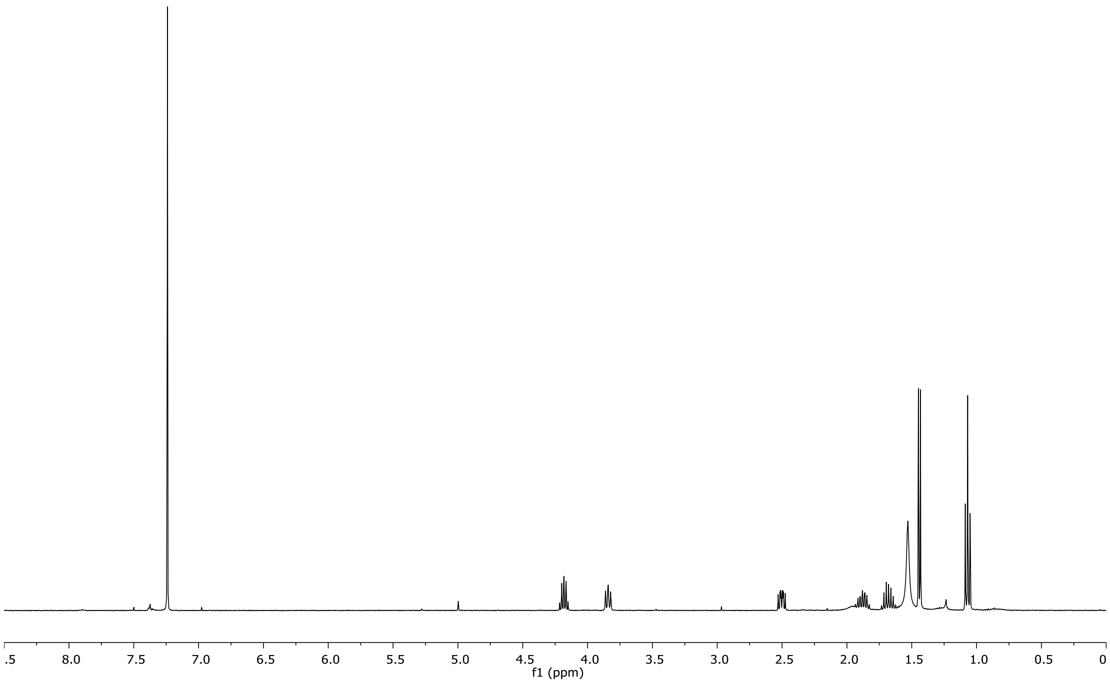

Figure S1. ^{1}H NMR spectrum ($CDCl_3$) of compound **1**.

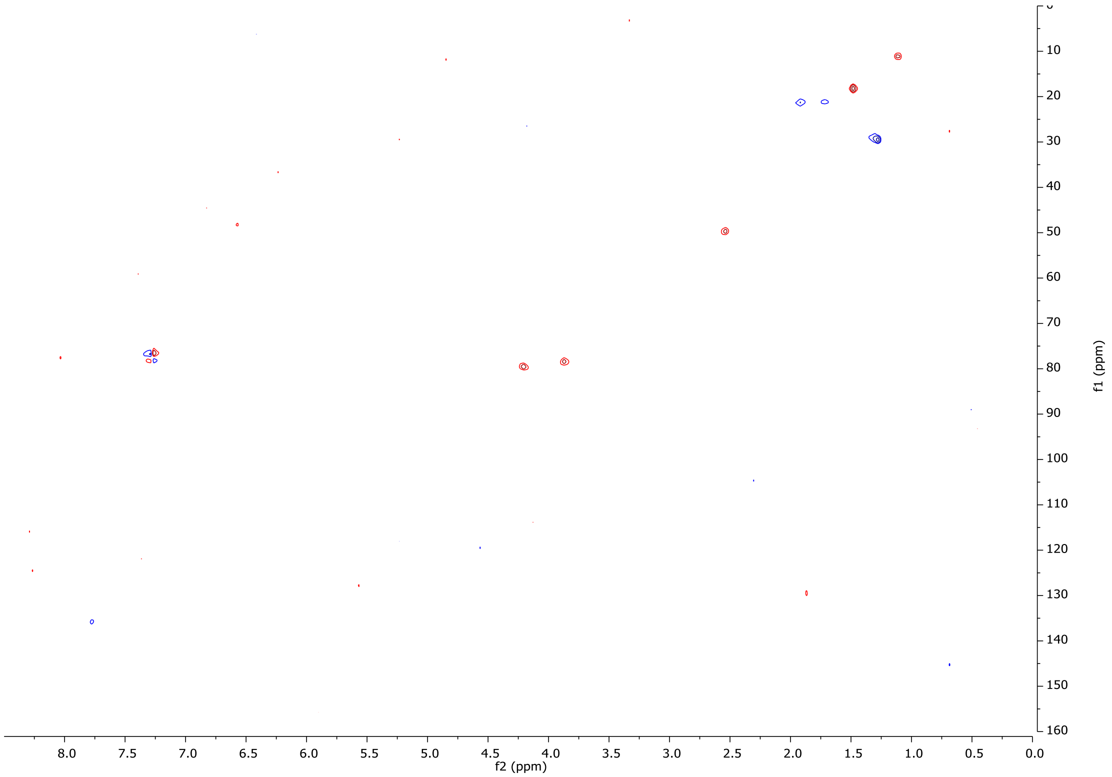

Figure S2. HSQC-DEPT spectrum ($CDCl_3$) of compound **1**.

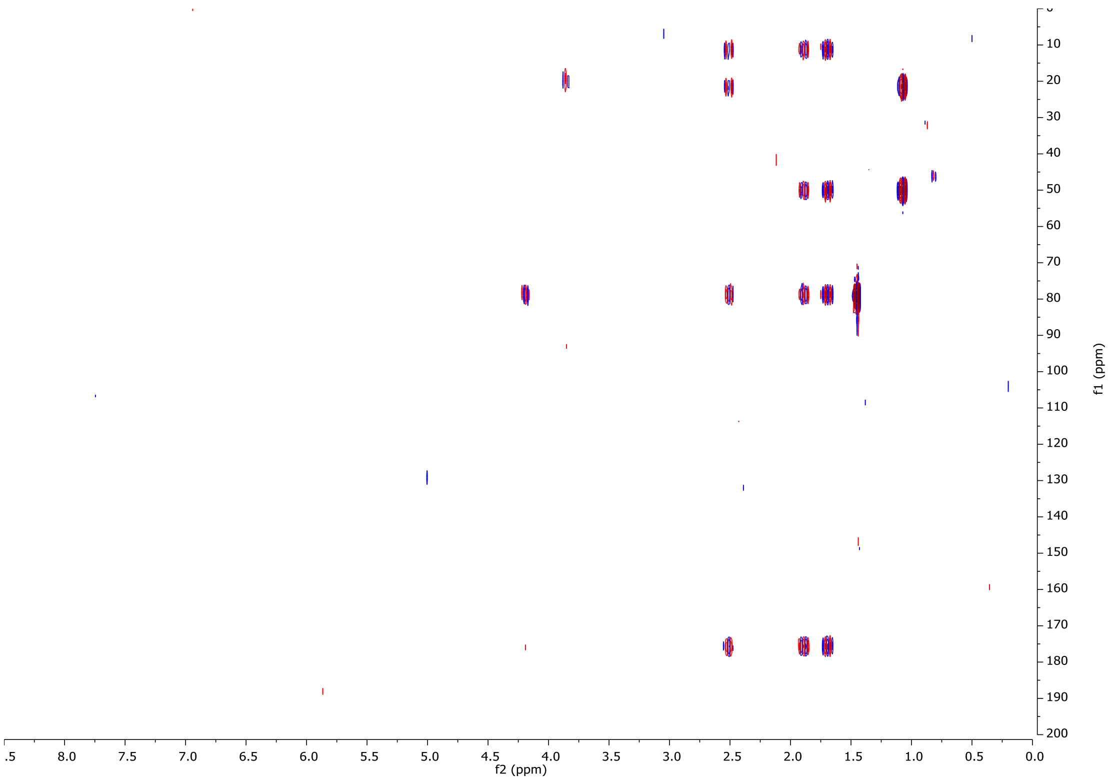

Figure S3. HMBC spectrum ($CDCl_3$) of compound **1**.

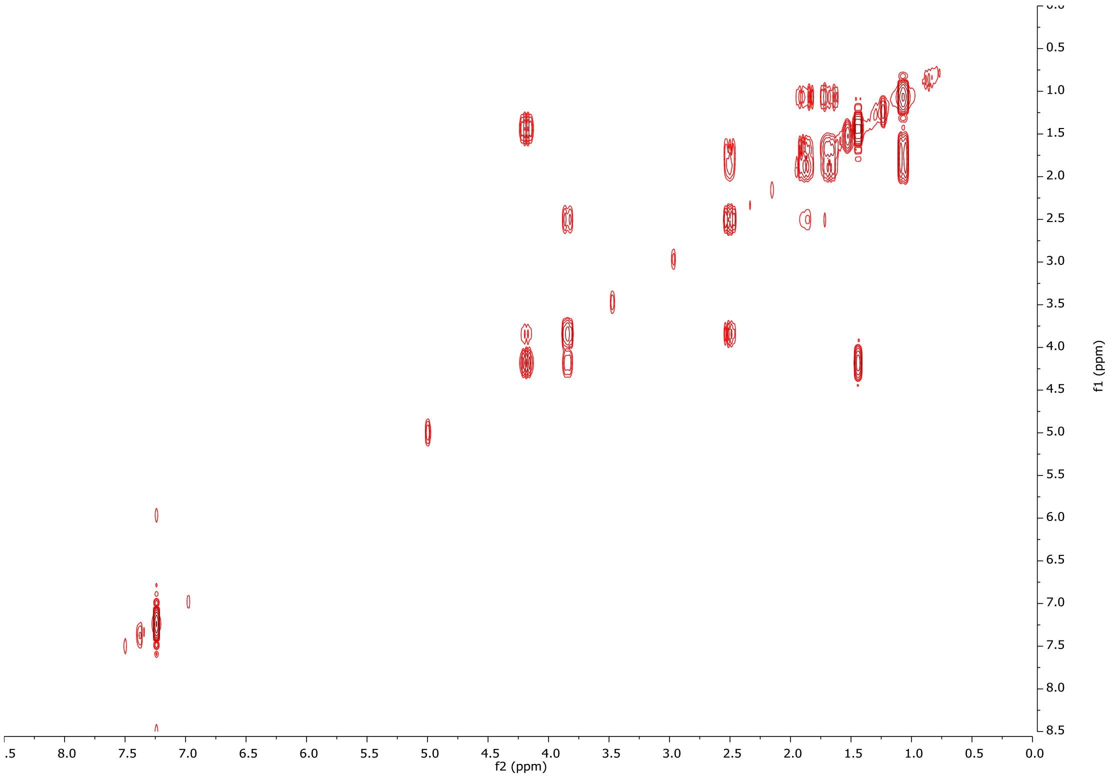

Figure S4. COSY spectrum ($CDCl_3$) of compound **1**.

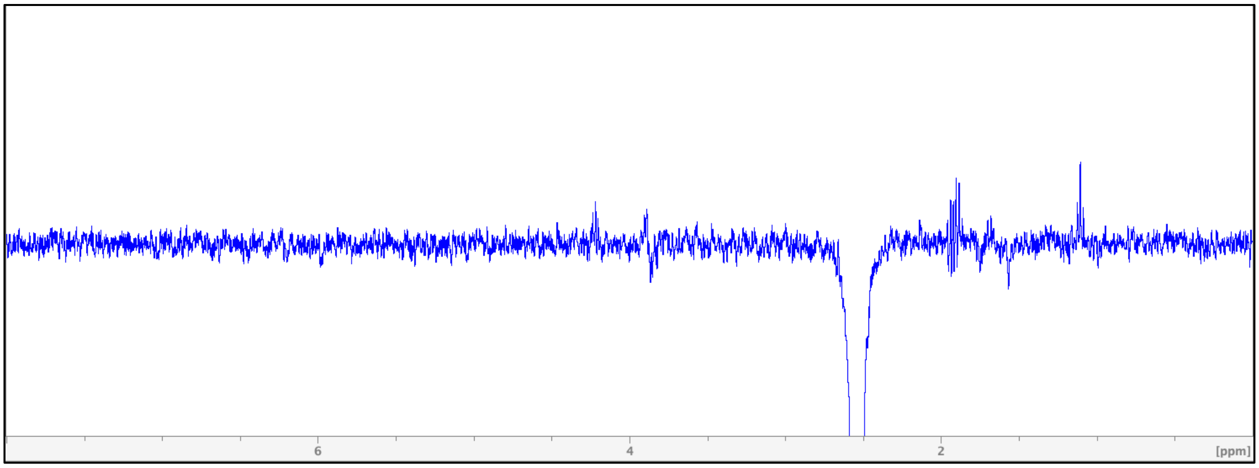

Figure S5. 1D NOE difference spectrum ($CDCl_3$) of compound **1** upon irradiation of H-2.

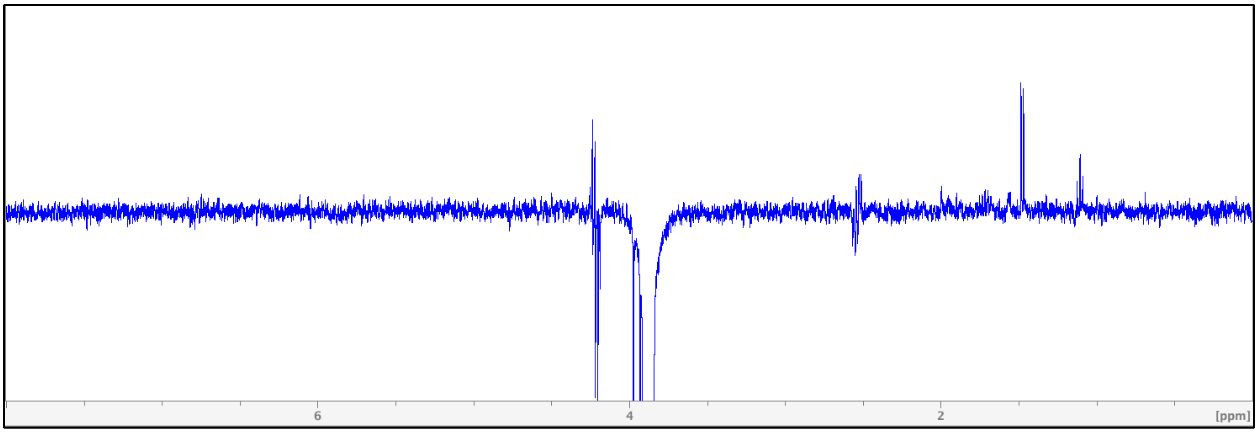

Figure S6. 1D NOE difference spectrum ($CDCl_3$) of compound **1** upon irradiation of H-3.

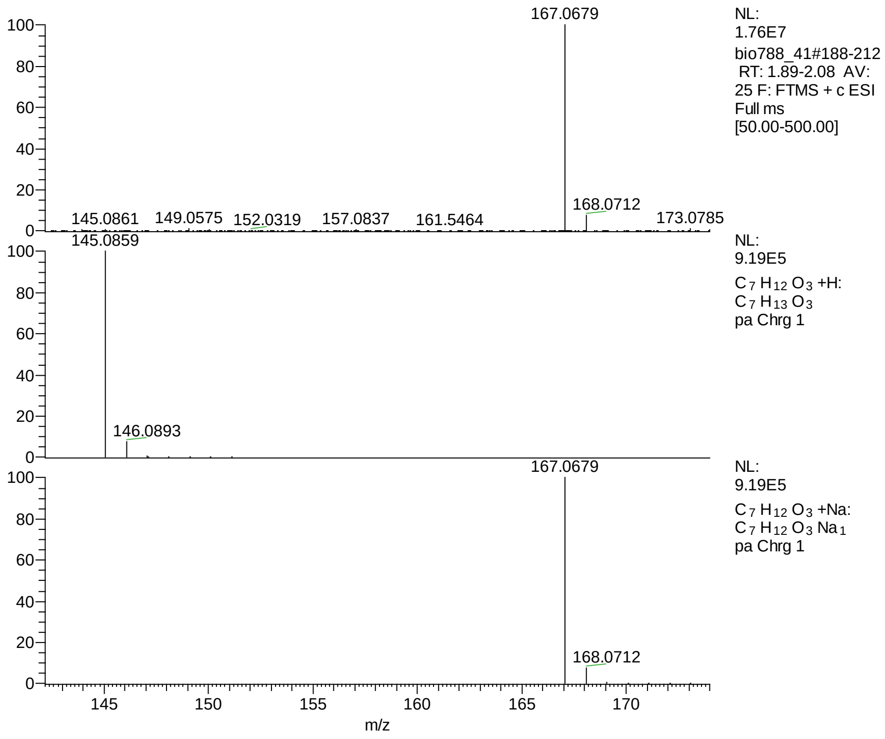

Figure S7. HR-ESIMS spectrum of compound **1**.

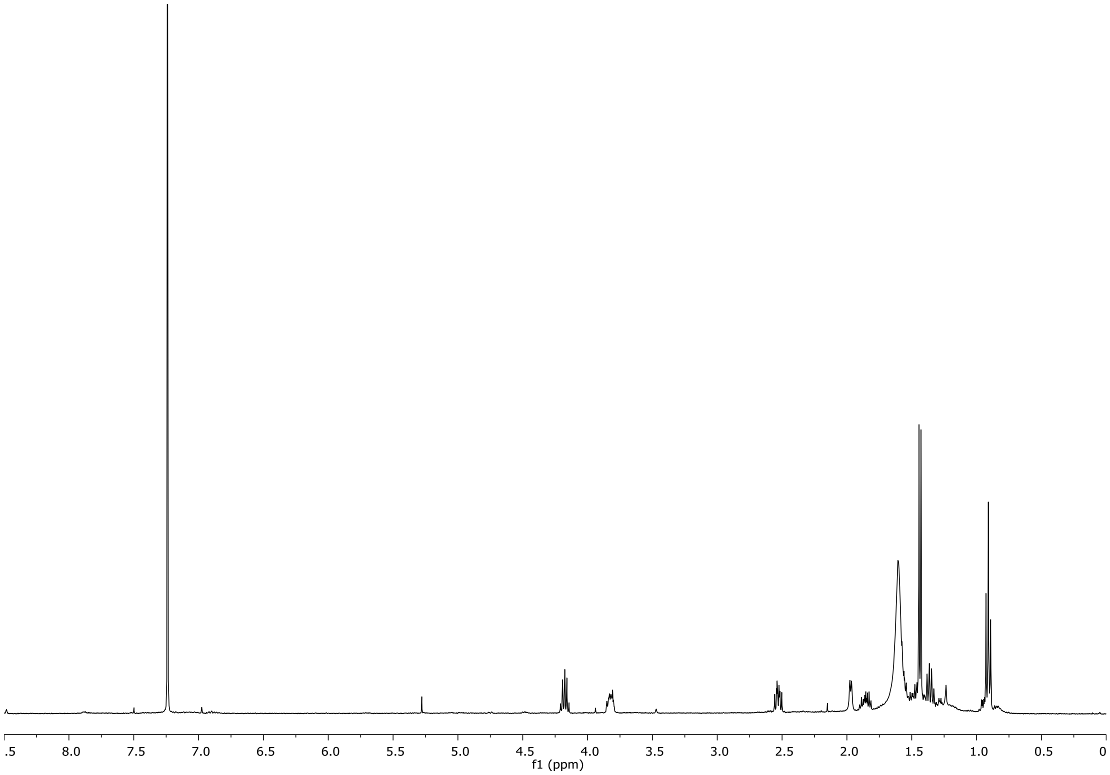

Figure S8. ^{1}H NMR spectrum ($CDCl_3$) of compound **2**.

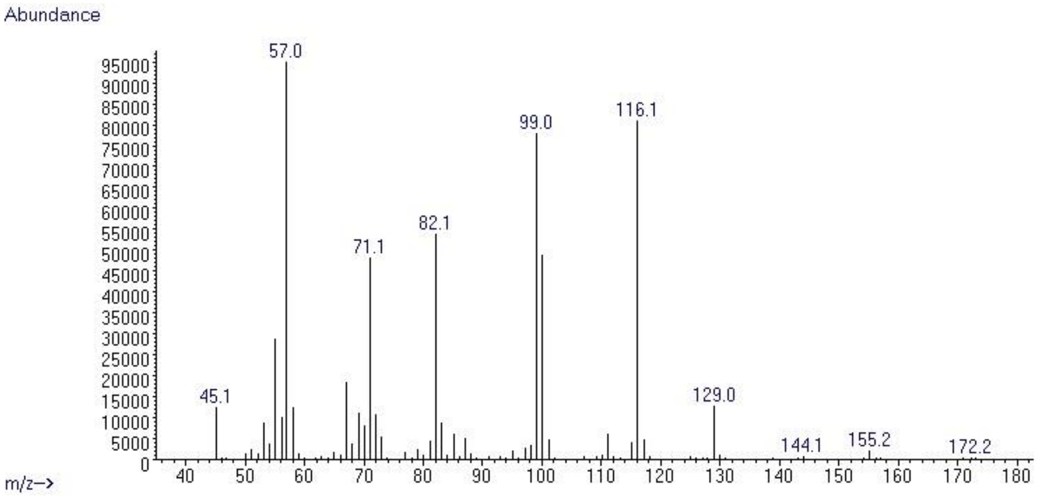

Figure S9. EIMS spectrum of compound **2**.

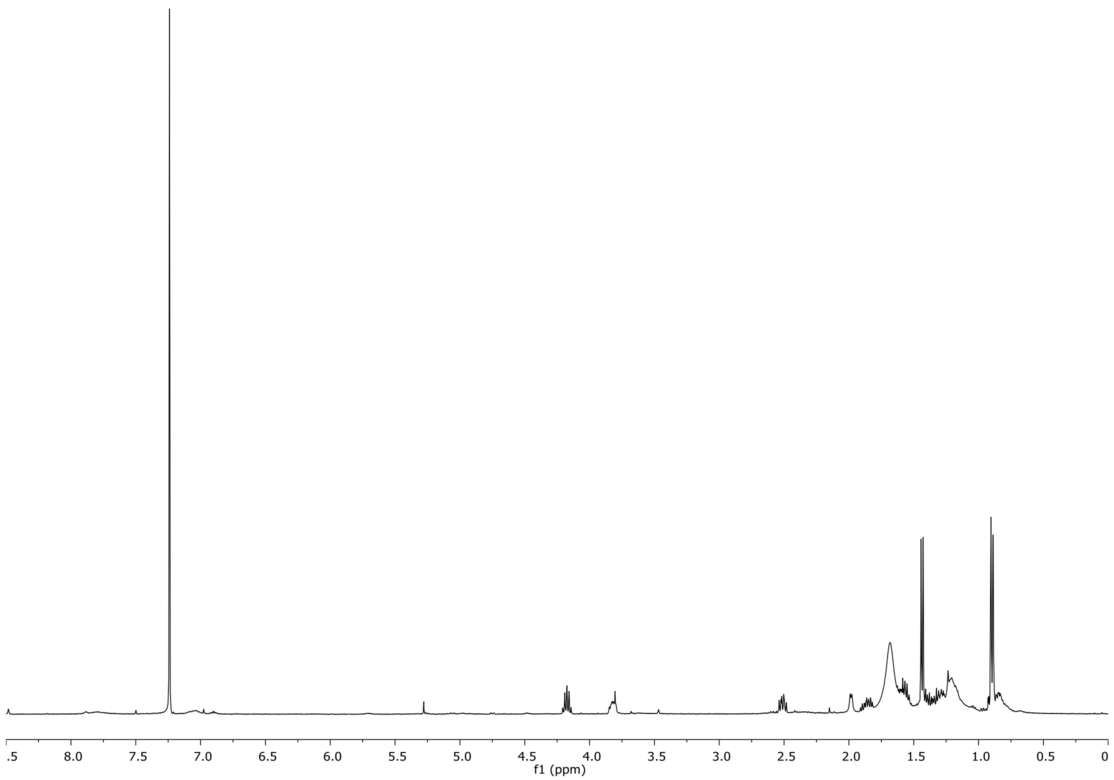

Figure S10. ^{1}H NMR spectrum ($CDCl_3$) of compound **3**.

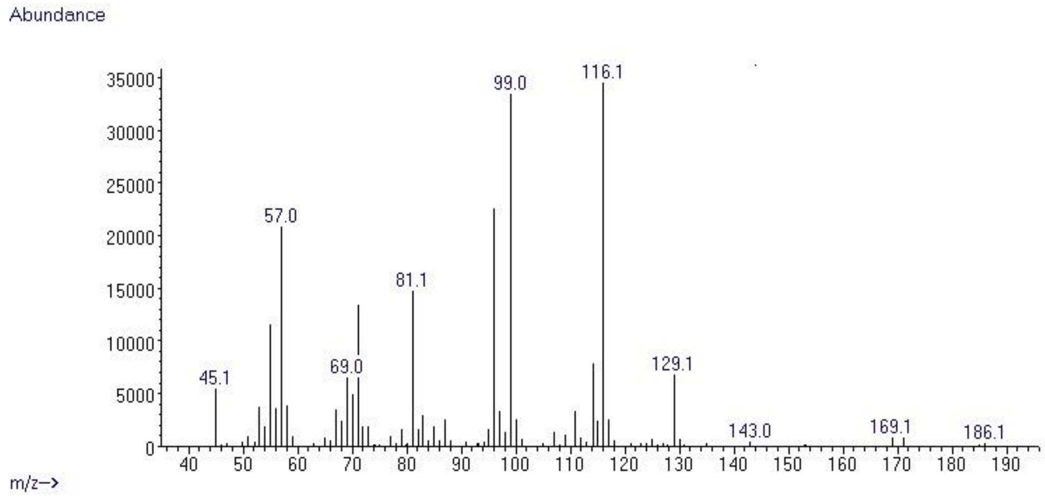

Figure S11. EIMS spectrum of compound **3**.

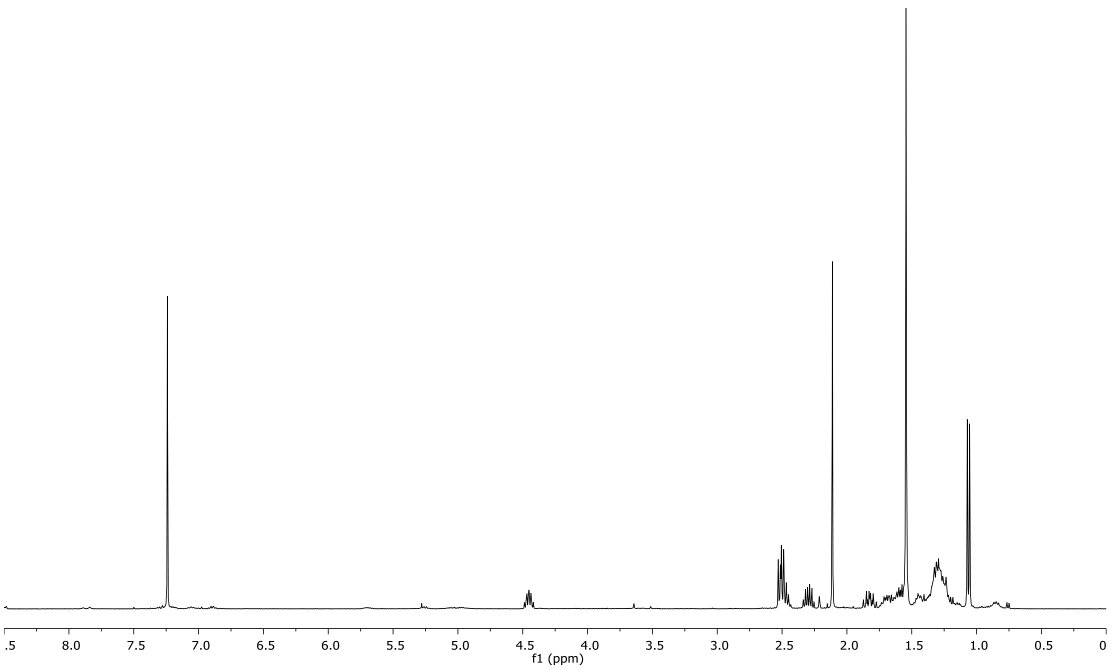

Figure S12. ^{1}H NMR spectrum ($CDCl_3$) of compound **4**.

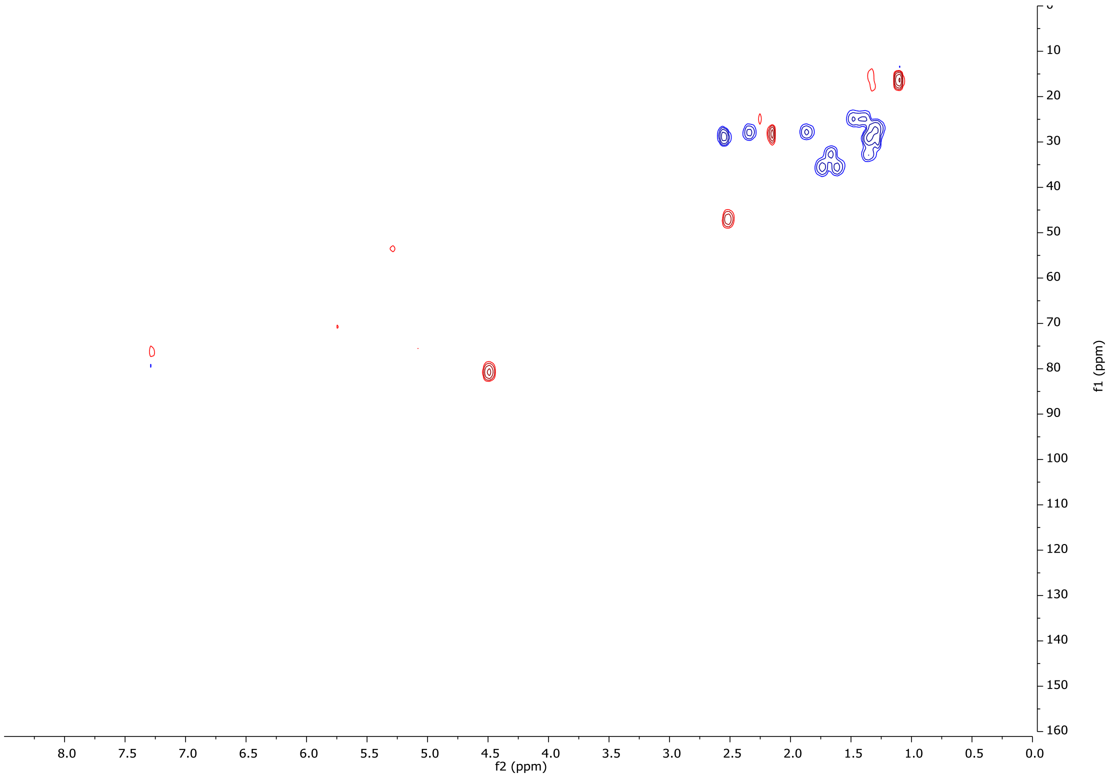

Figure S13. HSQC-DEPT spectrum ($CDCl_3$) of compound **4**.

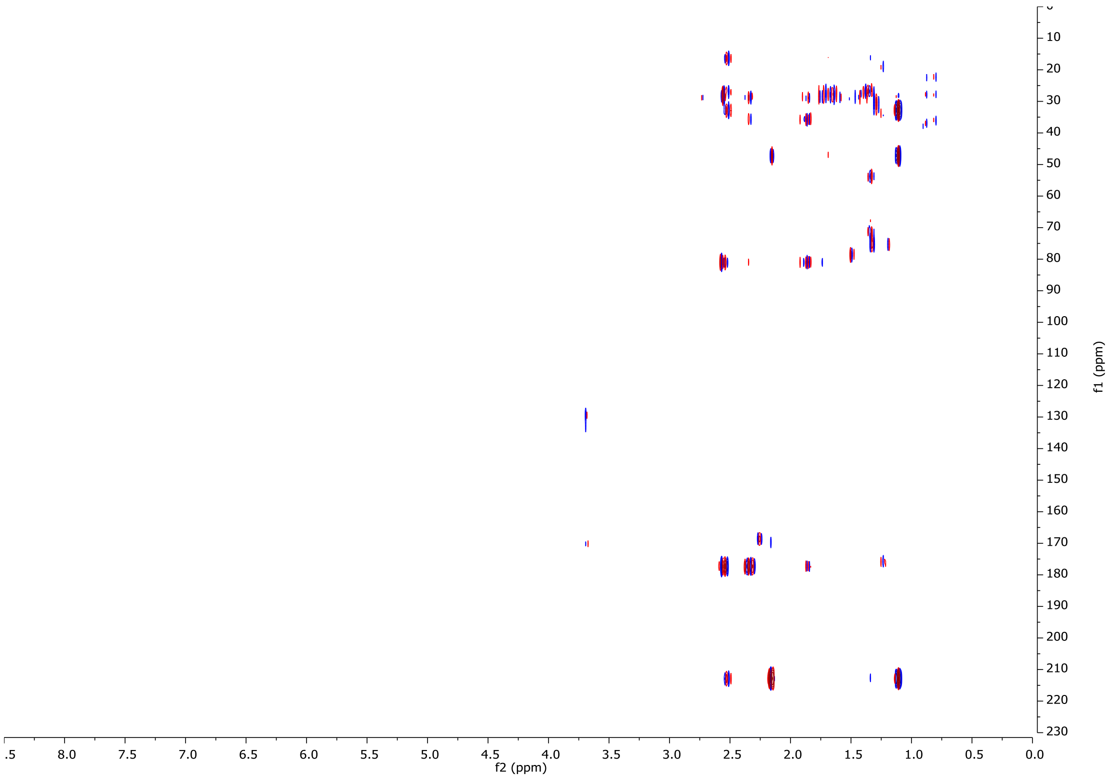

Figure S14. HMBC spectrum ($CDCl_3$) of compound **4**.

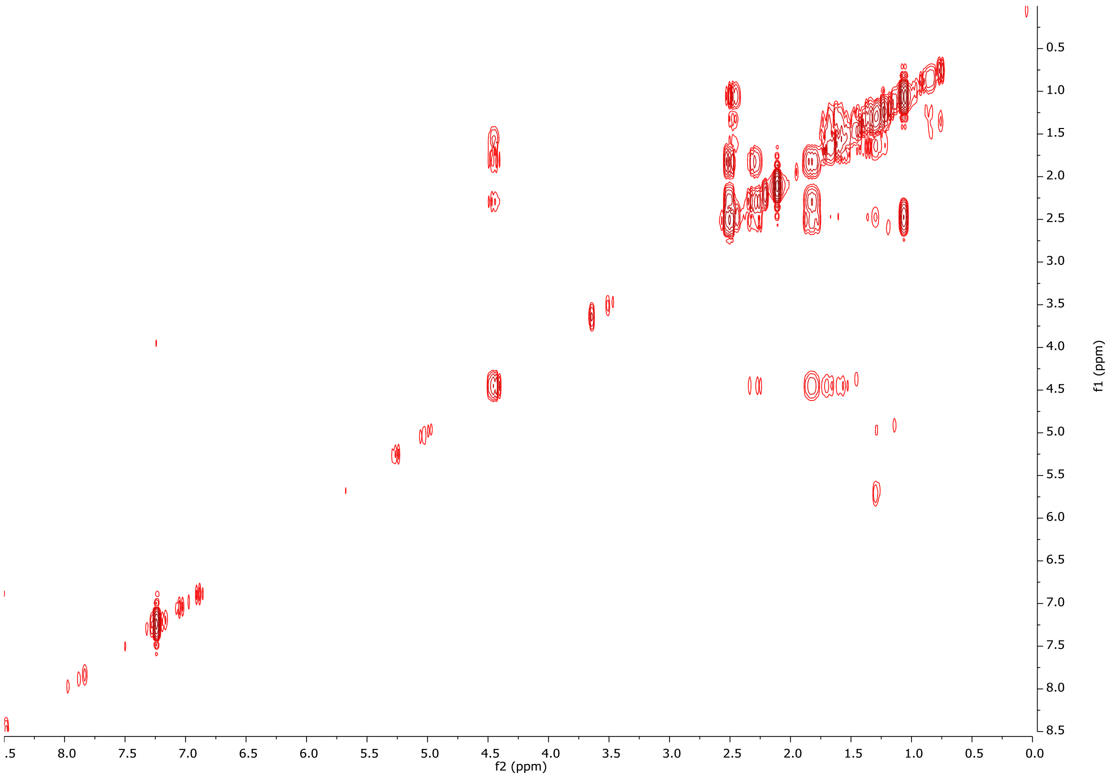

Figure S15. COSY spectrum ($CDCl_3$) of compound **4**.

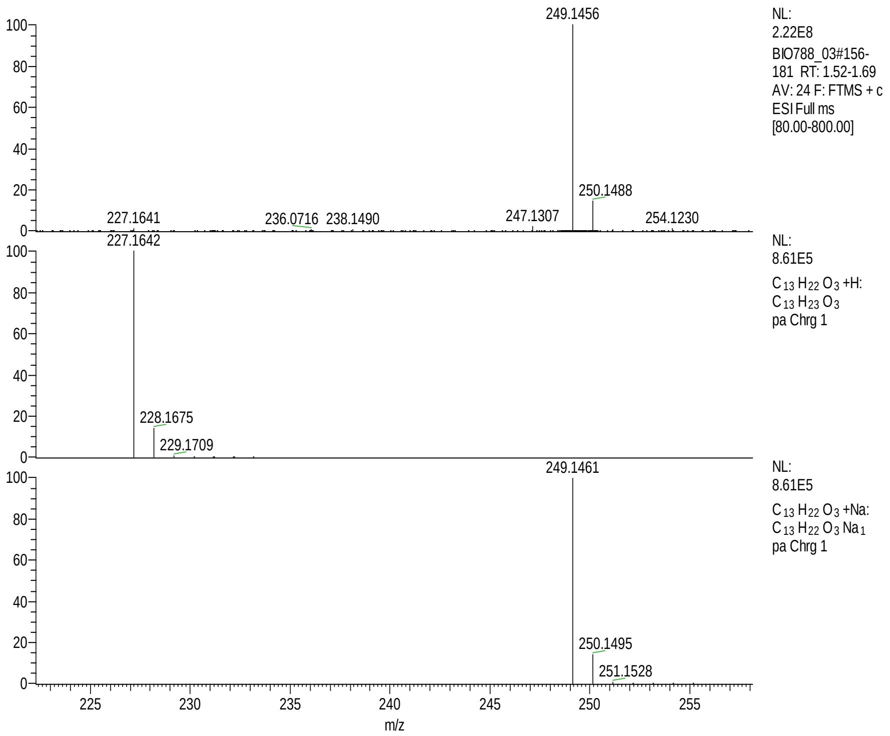

Figure S16. HR-ESIMS spectrum of compound **4**.

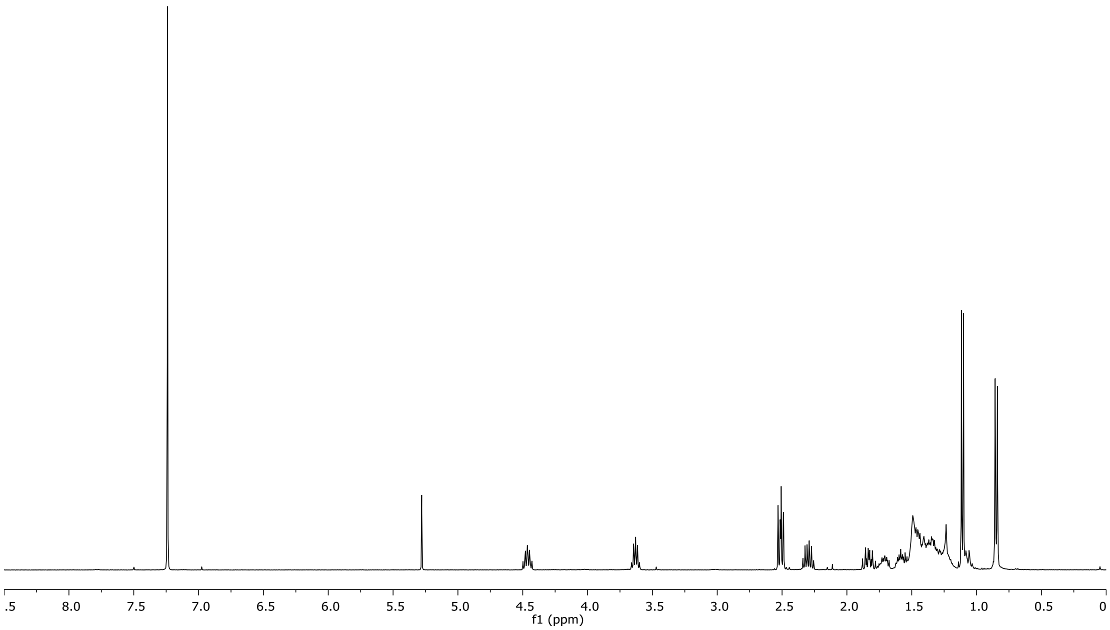

Figure S17. ^{1}H NMR spectrum ($CDCl_3$) of compound **5**.

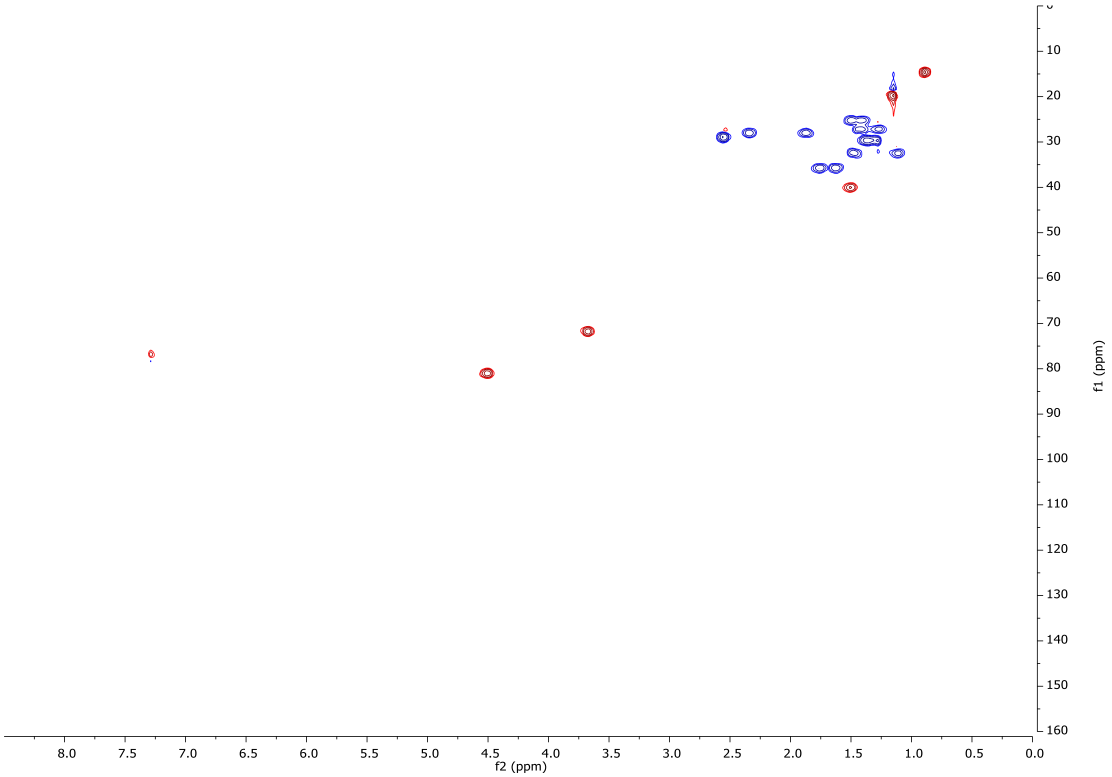

Figure S18. HSQC-DEPT spectrum ($CDCl_3$) of compound **5**.

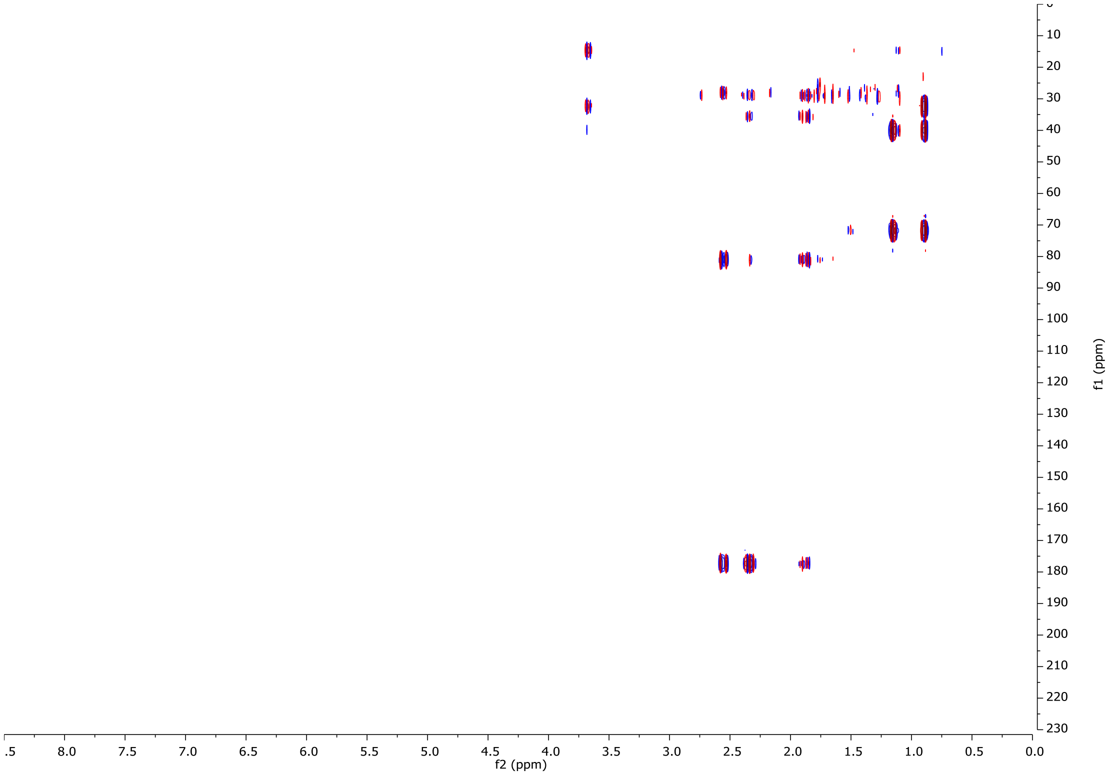

Figure S19. HMBC spectrum ($CDCl_3$) of compound **5**.

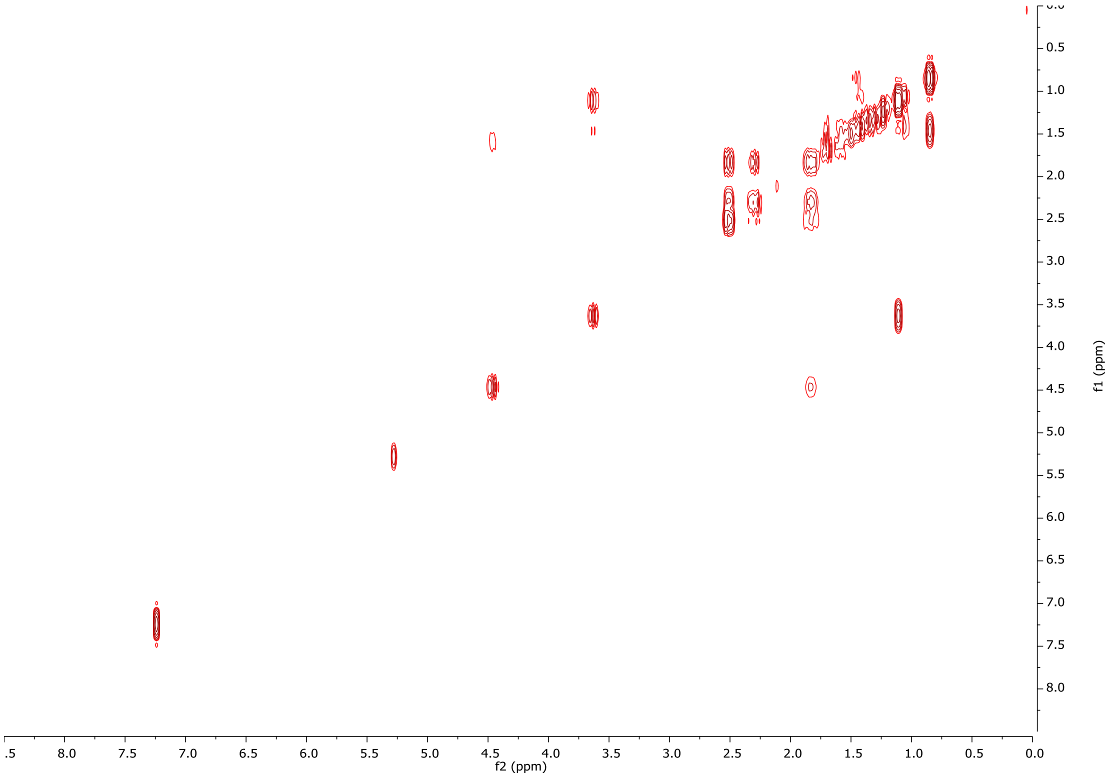

Figure S20. COSY spectrum ($CDCl_3$) of compound **5**.

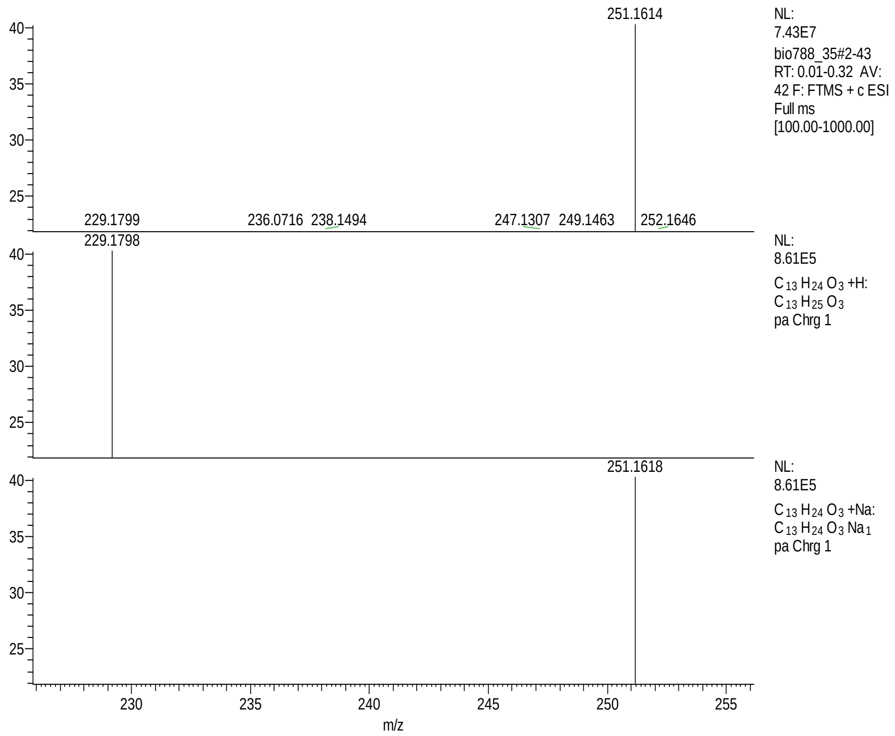

Figure S21. HR-ESIMS spectrum of compound **5**.

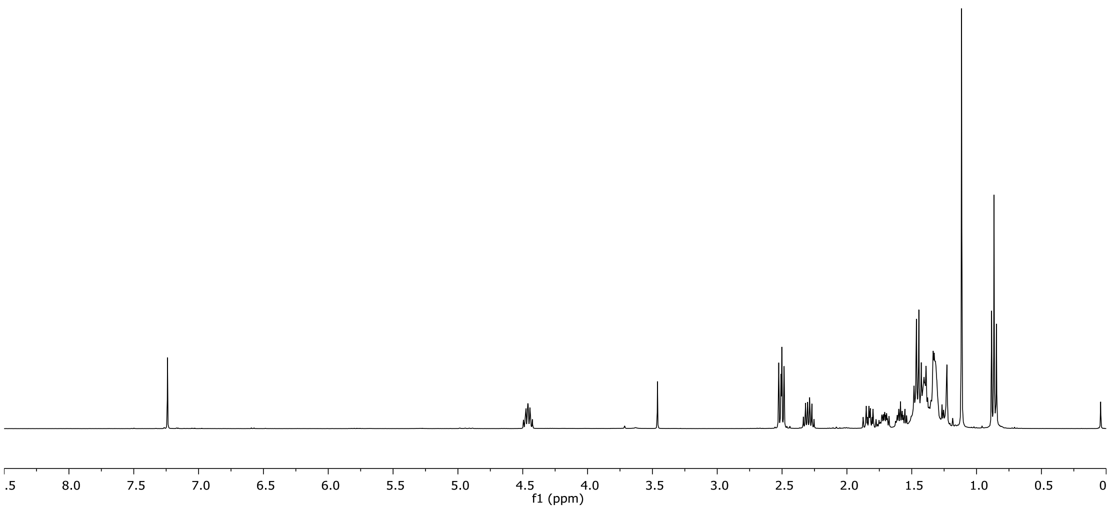

Figure S22. ^{1}H NMR spectrum ($CDCl_3$) of compound **6**.

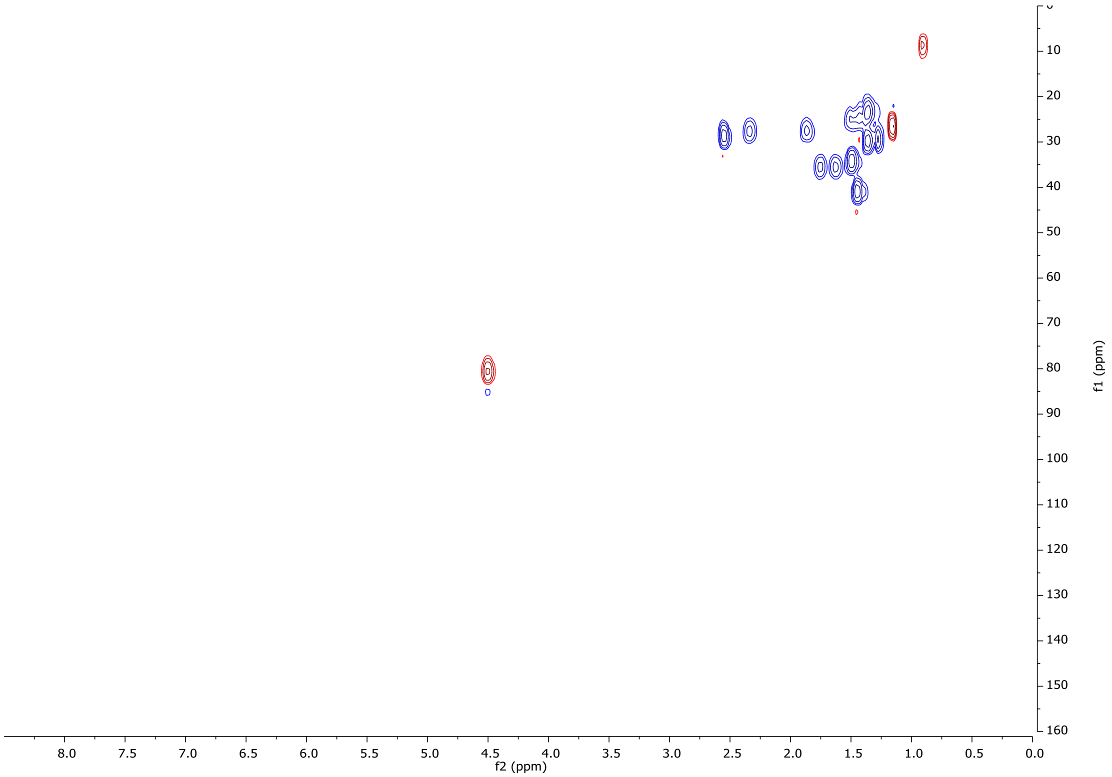

Figure S23. HSQC-DEPT spectrum ($CDCl_3$) of compound **6**.

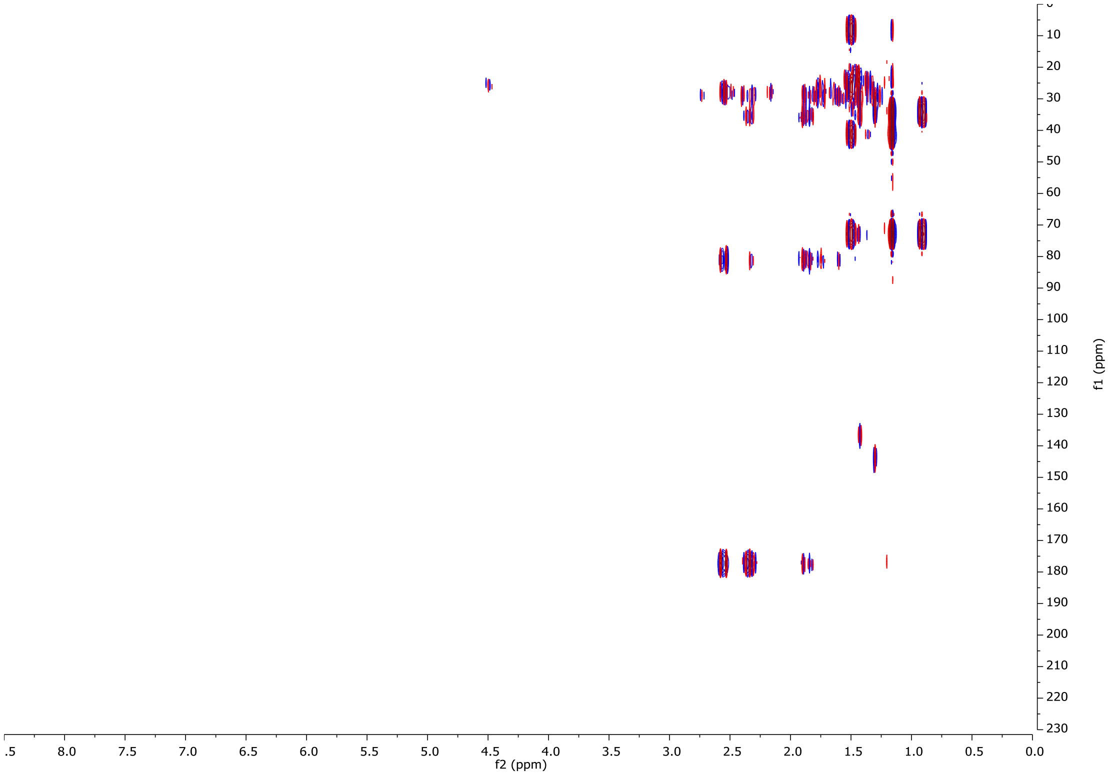

Figure S24. HMBC spectrum (CDCl3) of compound **6**.

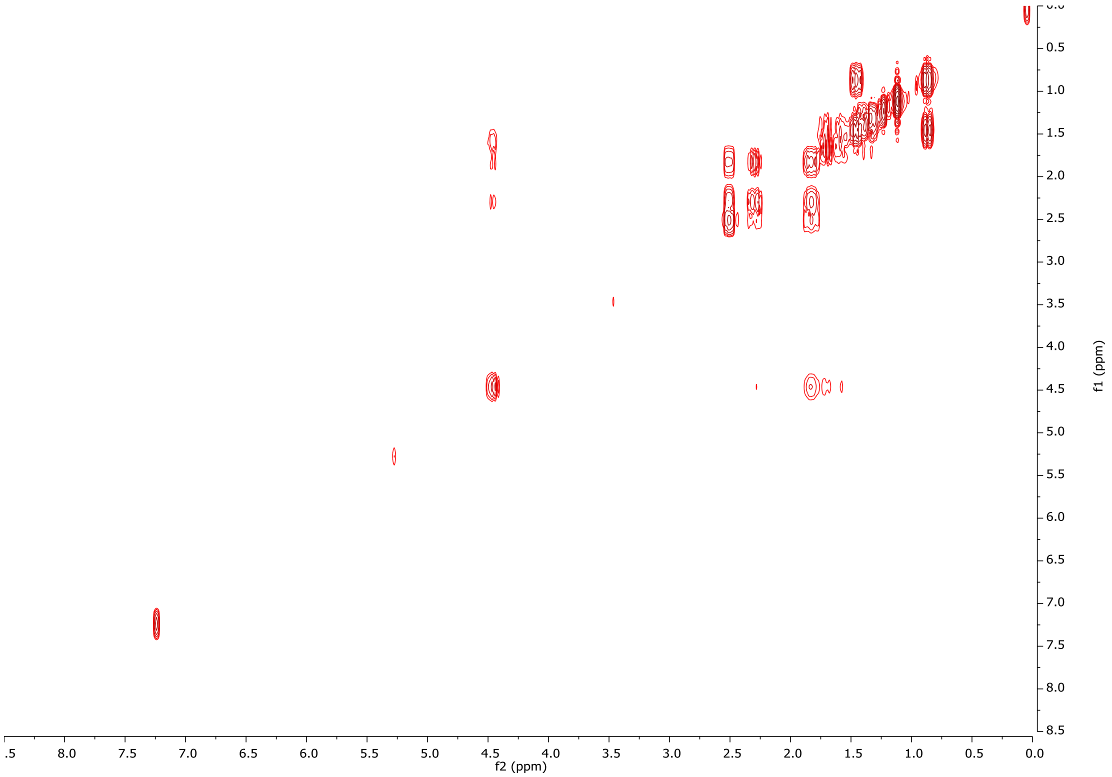

Figure S25. COSY spectrum (CDCl3) of compound **6**.

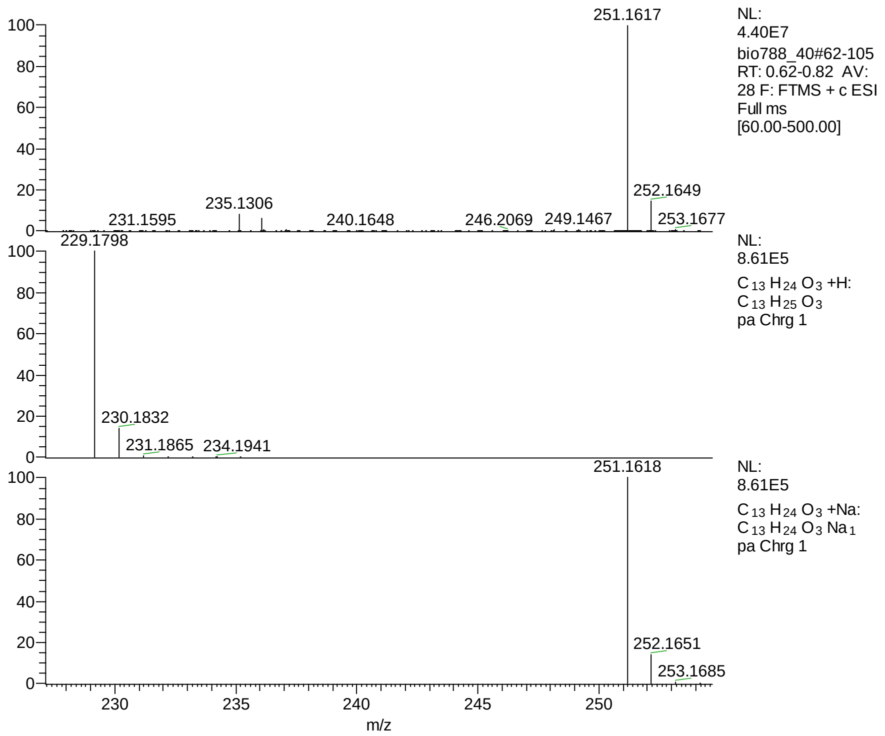

Figure S26. HR-ESIMS spectrum of compound **6**.

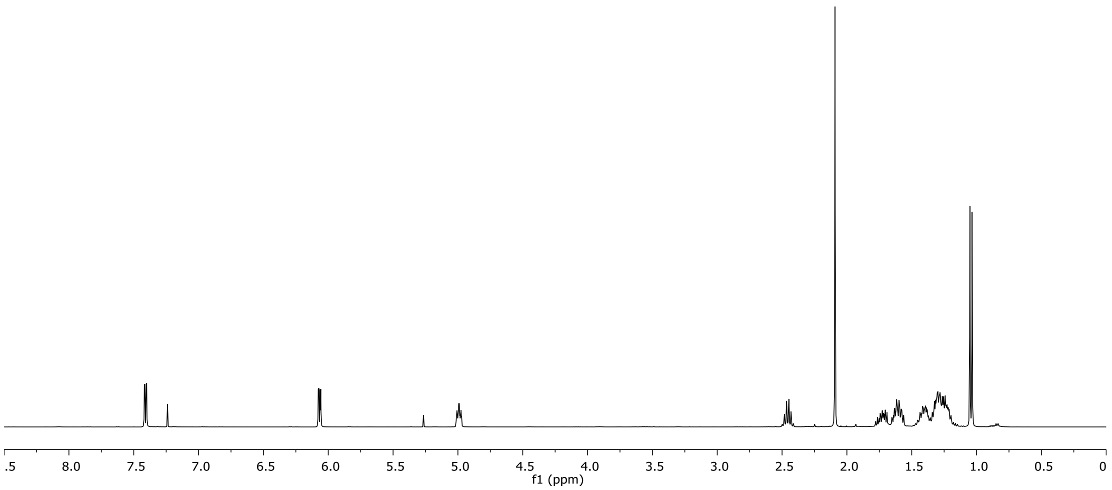

Figure S27. ^{1}H NMR spectrum ($CDCl_3$) of compound **7**.

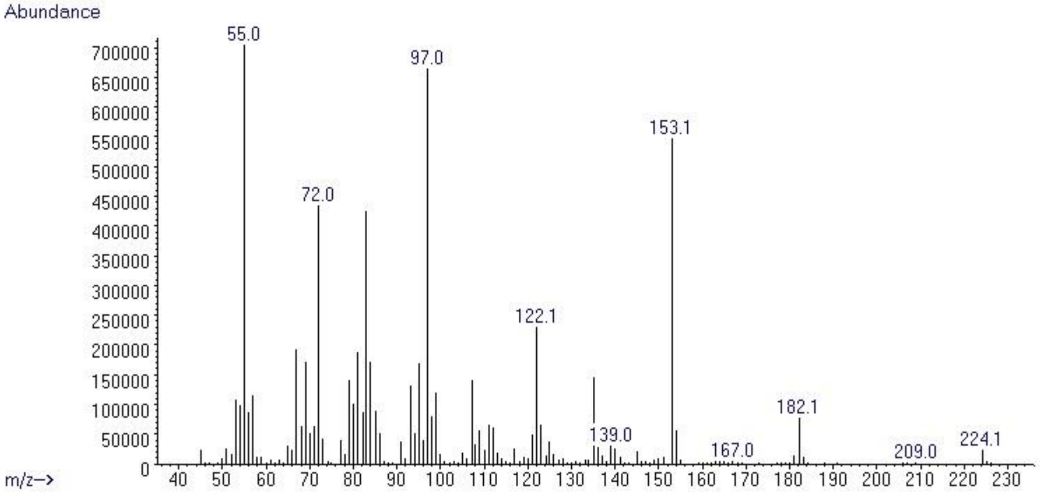

Figure S28. EIMS spectrum of compound **7**.

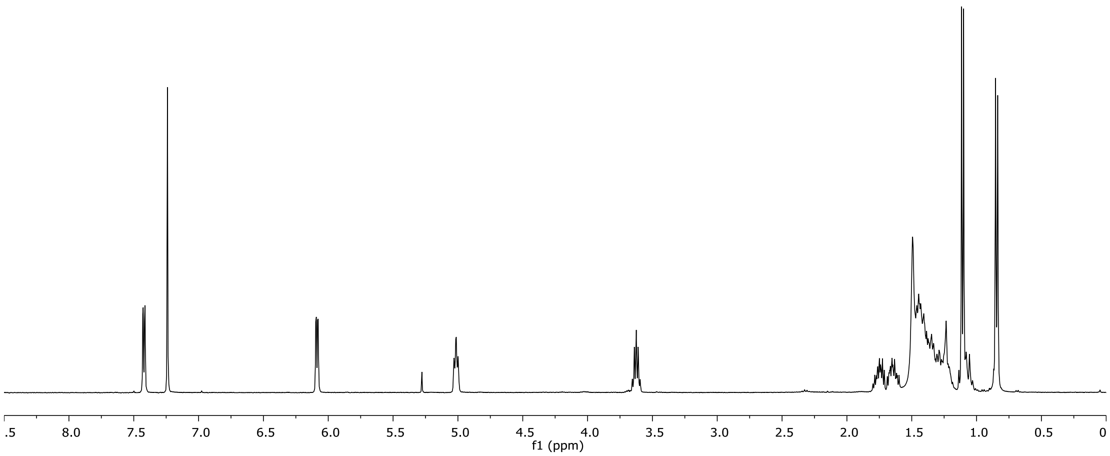

Figure S29. ^{1}H NMR spectrum ($CDCl_3$) of compound **8**.

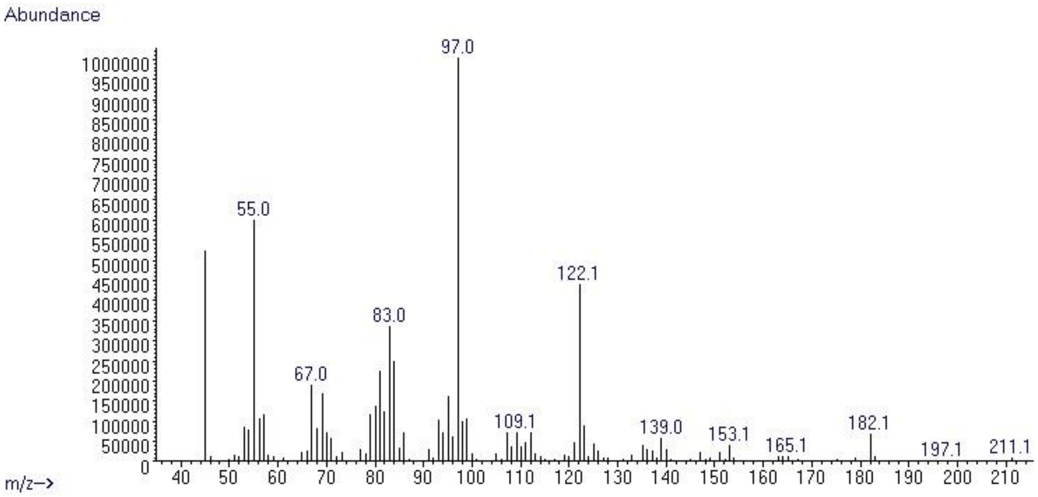

Figure S30. EIMS spectrum of compound **8**.

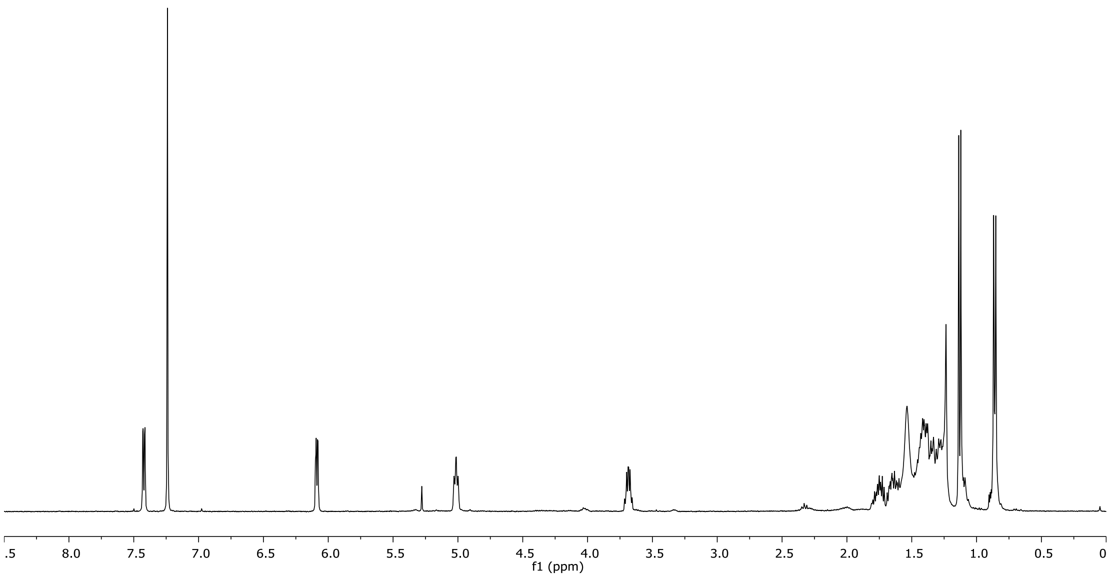

Figure S31. ¹H NMR spectrum ($CDCl_3$) of compound **9**.

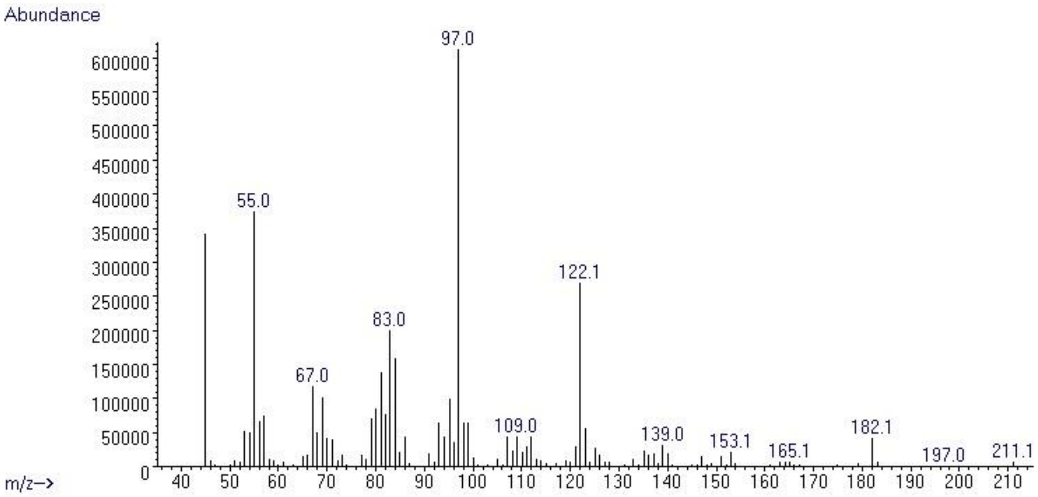

Figure S32. EIMS spectrum of compound **9**.

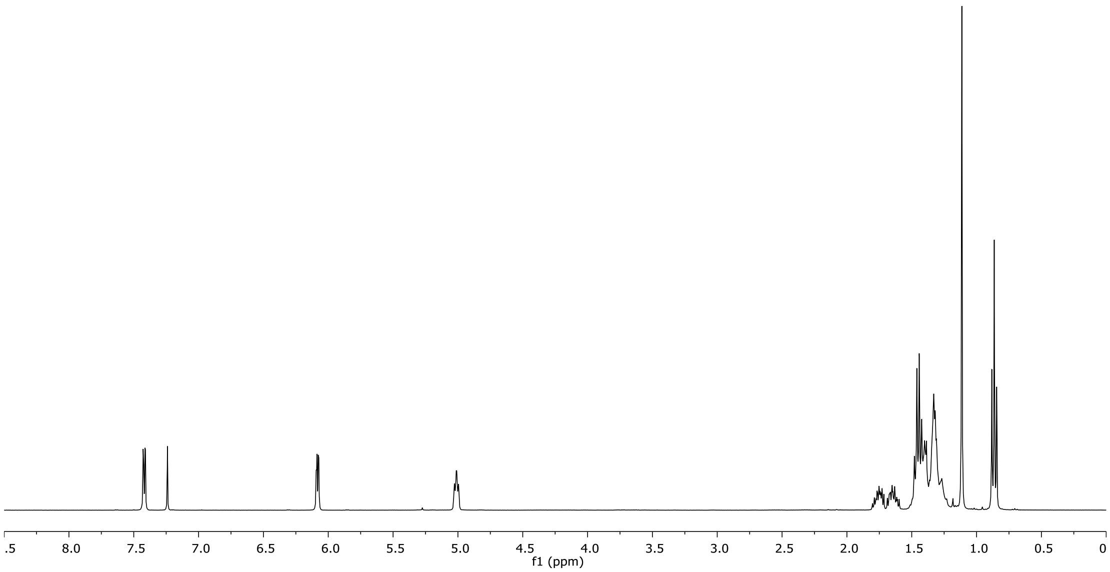

Figure S33. ^{1}H NMR spectrum ($CDCl_3$) of compound **10**.

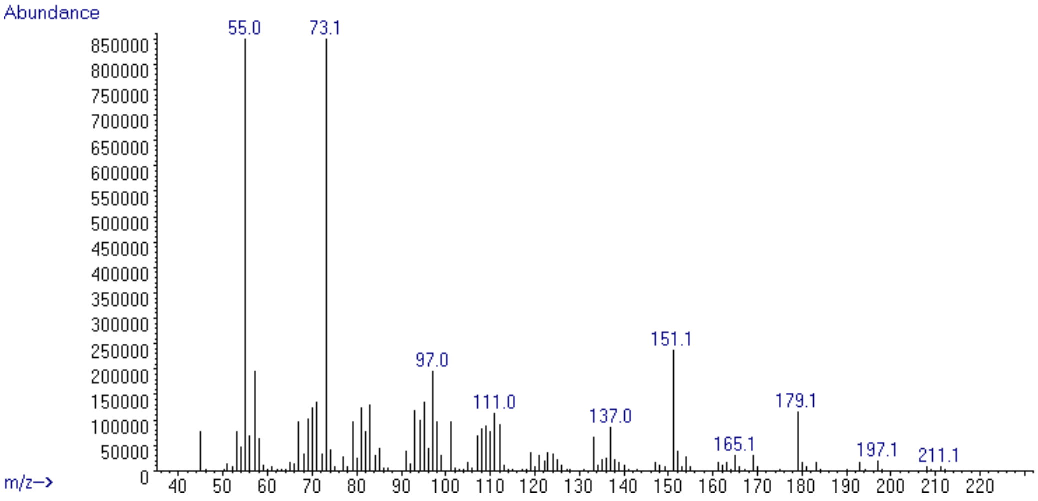

Figure S34. EIMS spectrum of compound **10**.

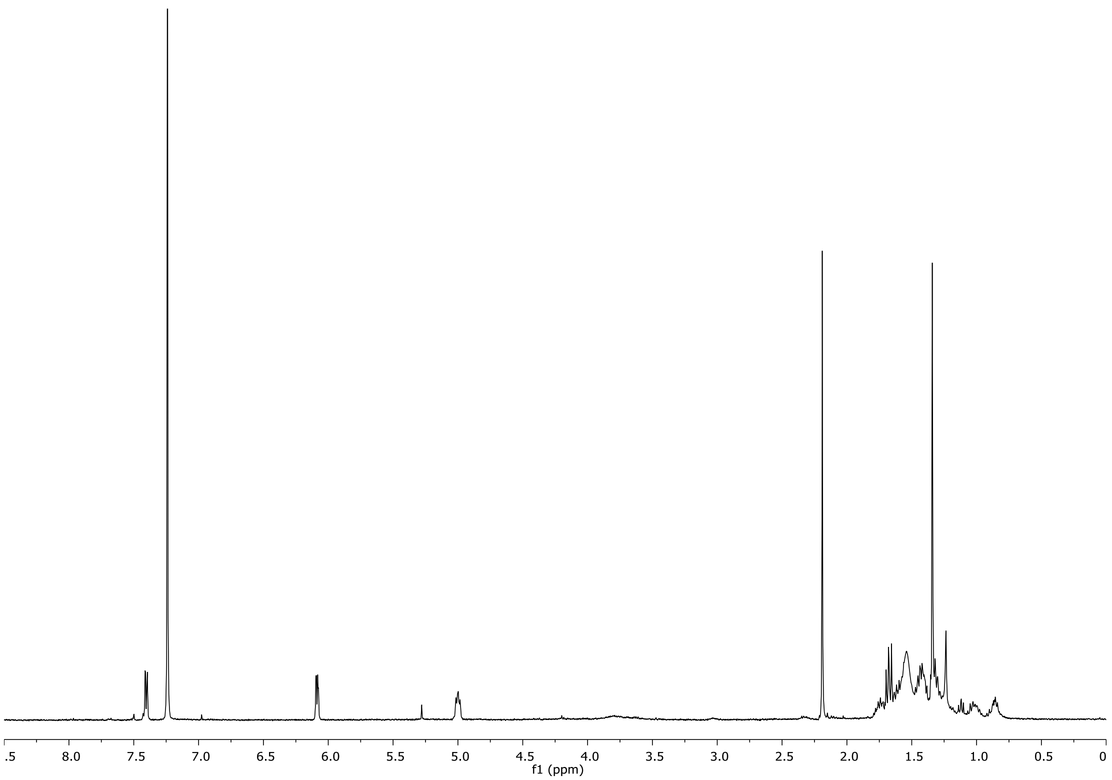

Figure S35. ^{1}H NMR spectrum ($CDCl_3$) of compound **11**.

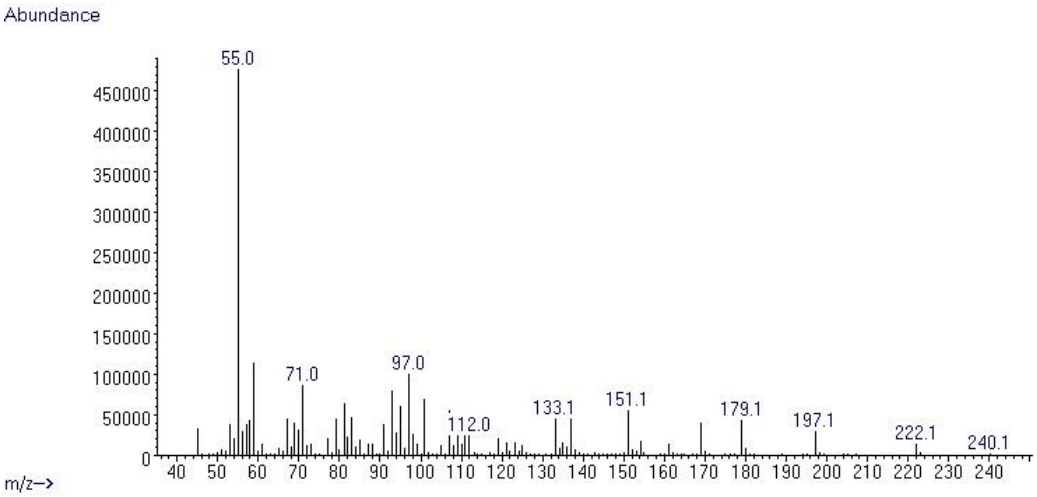

Figure S36. EIMS spectrum of compound **11**.

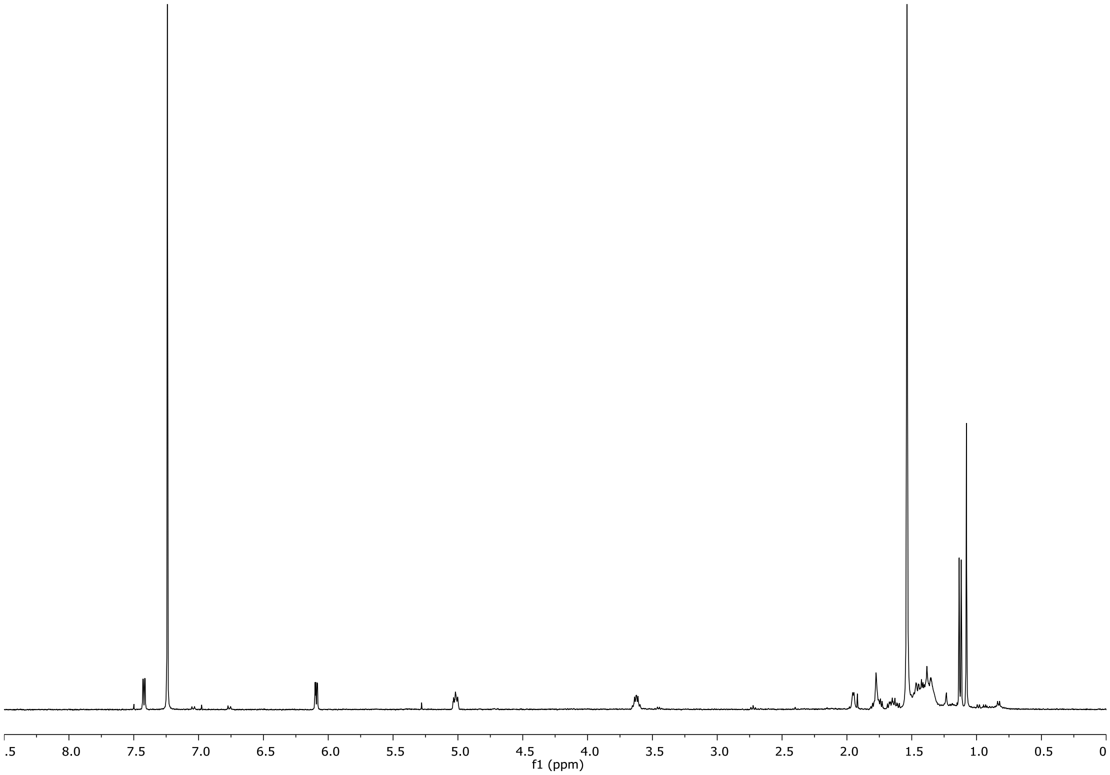

Figure S37. ^1H NMR spectrum ($CDCl_3$) of compound **12**.

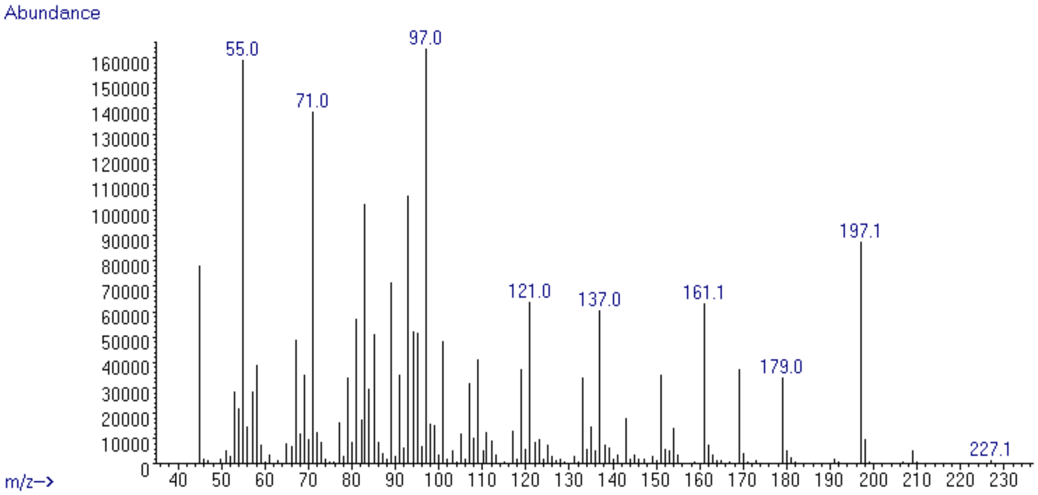

Figure S38. EIMS spectrum of compound **12**.

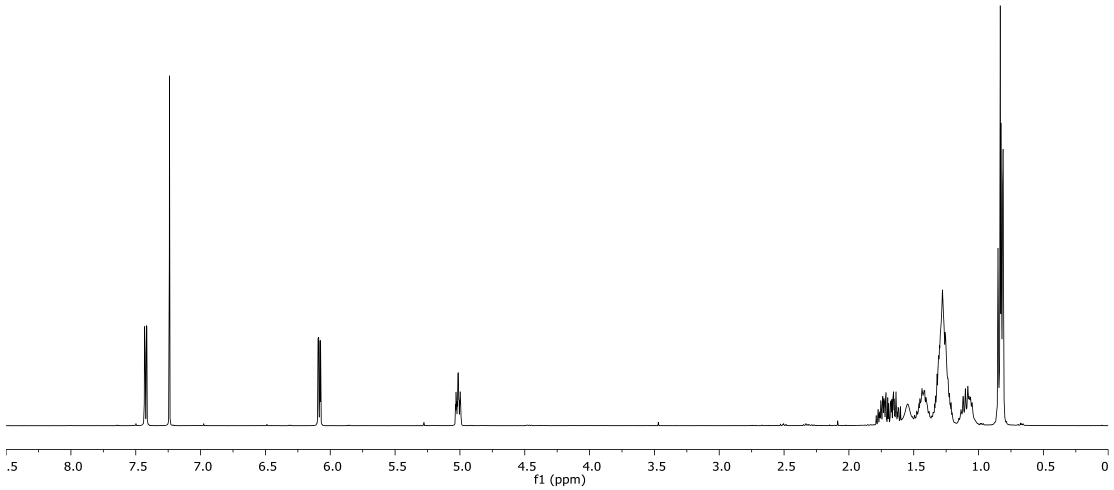

Figure S39. ^1H NMR spectrum ($CDCl_3$) of compound **13**.

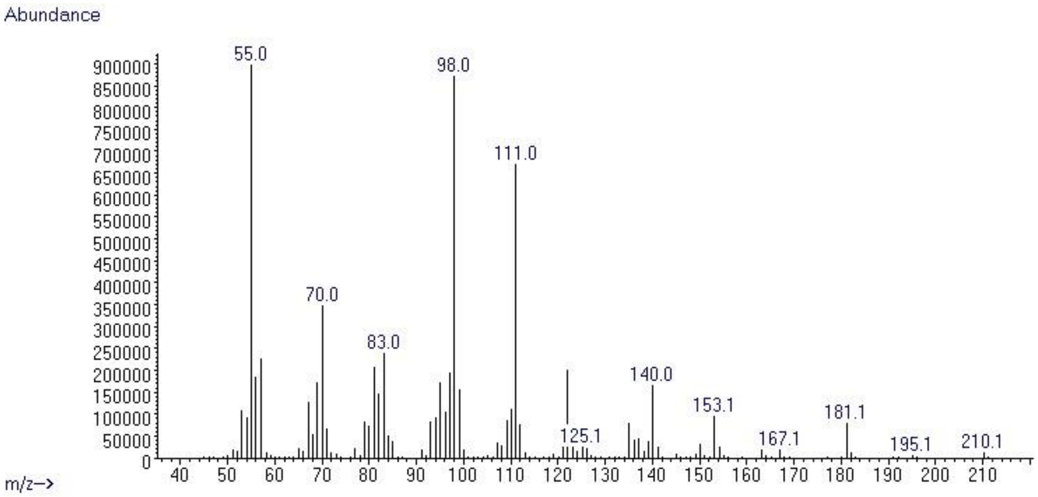

Figure S40. EIMS spectrum of compound **13**.

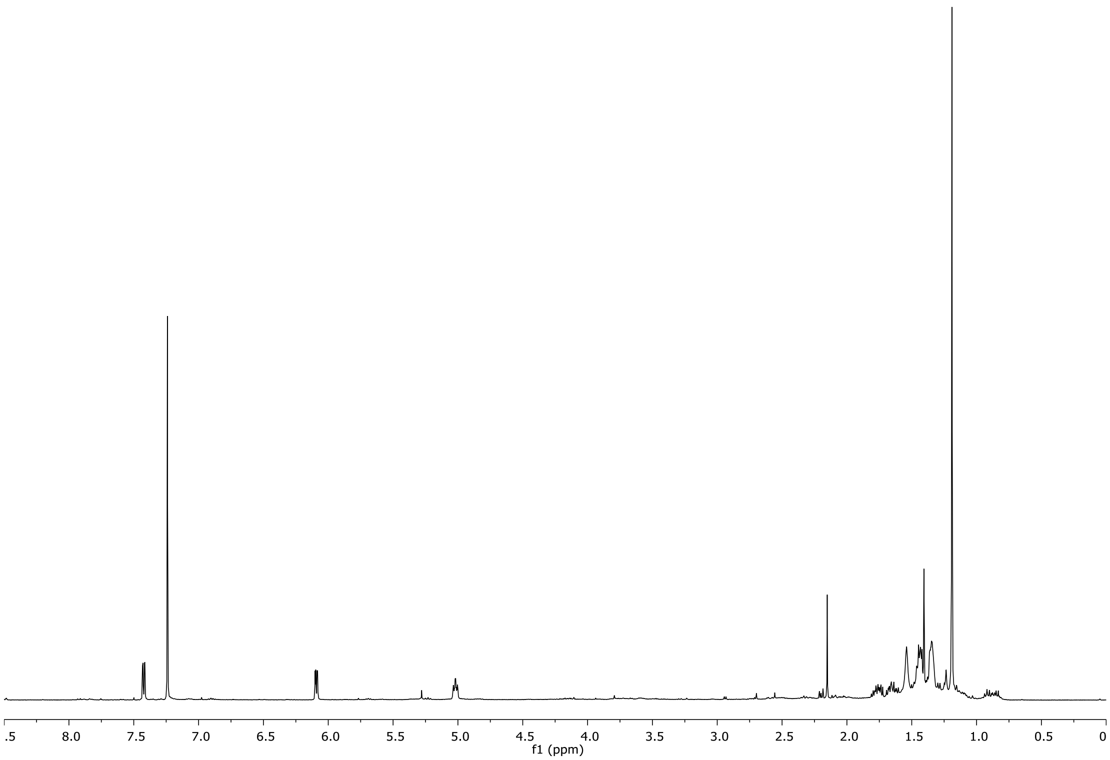

Figure S41. ^{1}H NMR spectrum ($CDCl_3$) of compound **14**.

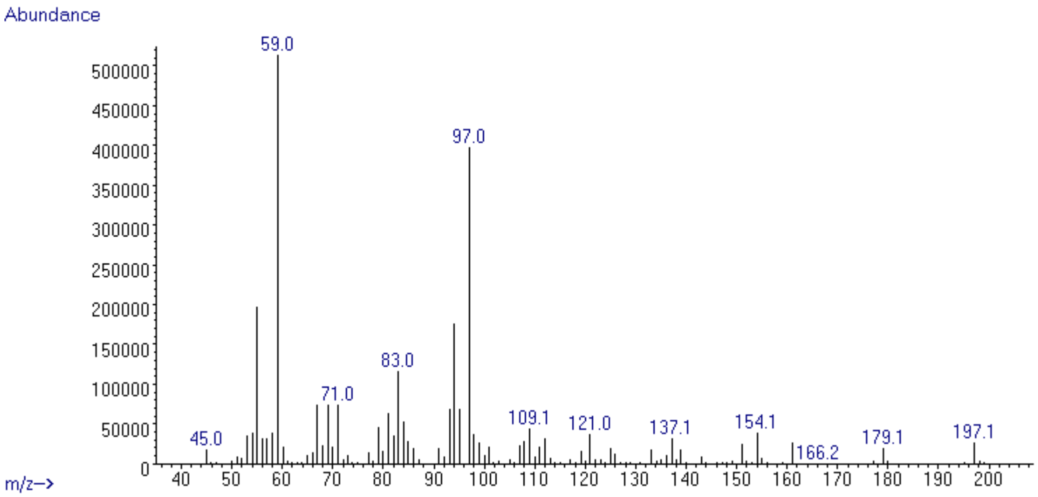

Figure S42. EIMS spectrum of compound **14**.
